# Bioprinting of Nanocellulose Hydrogels for Photobiocatalysis Under Continuous Flow

**DOI:** 10.1021/acssuschemeng.5c09689

**Published:** 2026-04-01

**Authors:** Lenny Malihan-Yap, Lisa Schmedler, Daniel Pint, Hitesh Medipally, Florian Lackner, Simon Fedrigotti, Rupert Kargl, Karin Stana Kleinschek, Robert Kourist, Heidrun Gruber-Woelfler

**Affiliations:** † Institute of Molecular Biotechnology, 27253 Graz University of Technology, Petersgasse 14, 8010 Graz, Austria; ‡ Institute of Process and Particle Engineering, Graz University of Technology, Inffeldgasse 13, 8010 Graz, Austria; § Institute of Chemistry and Technology of Biobased System (IBioSys) Graz University of Technology, Stremayrgasse 9, 8010 Graz, Austria

**Keywords:** 3D printing, cyanobacteria, biotransformation, immobilization, continuous flow, (bio)reactor engineering, photobiocatalysis

## Abstract

Photosynthetic microorganisms are capable of oxygenic photosynthesis, delivering both oxygen and cofactors to drive enzymatic redox reactions. However, their dependence on visible light limits the tolerable cell densities to achieve high reaction rates. Immobilizing cells within a matrix often increases biocatalyst productivity while allowing facile retainment but also creates mass transfer limitations across the solid–liquid interface. Herein, we address these challenges and present the immobilization of recombinant cyanobacteria in 3D-printed hydrogels of varying geometries. In particular, whole cells of the cyanobacterium *Synechocystis* sp. PCC 6803, engineered to express the gene of the ene-reductase YqjM, were implemented in biocompatible hydrogels made out of nanofibrillated cellulose and alginate. The hydrogels were 3D-printed via extrusion into different geometries to alleviate light and mass transfer limitations and were applied for the reduction of prochiral 2-methylmaleimide to (*R*)-2-methylsuccinimide. The obtained reactors exhibit high mechanical stability (620 kPa), efficient flow and mass transfer characteristics, high specific surface area (up to 2129 mm^2^ g^–1^), and retention times favorable to achieve high product formation. (*R*)-2-methylsuccinimide was obtained with a space–time yield of 0.28 g L^–1^ h^–1^ and a high enantiomeric purity (>99%). The highly atom-efficient chemical process (88%) using only water to provide electrons for NADPH regeneration could be upscaled and can potentially be operated in extended periods to reduce wastewater associated with cell cultivation. Overall, 3D printing of photosynthetic microorganisms embedded in a hydrogel matrix holds significant promise for advancing the development of whole-cell solid-state photosynthetic cell factories. These are important steps toward improved reactor designs and higher efficiencies to improve crucial redox biotransformations.

## Graphical abstract

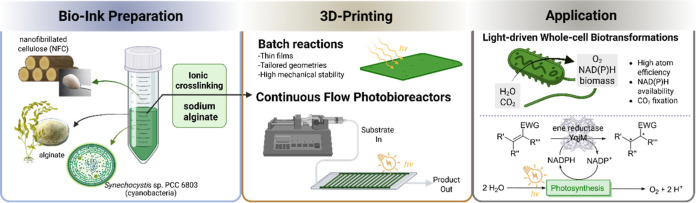

## 1 Introduction

Three-dimensional (3D) printing, also known as additive manufacturing, has emerged as a versatile and effective technology with various applications in biotechnology, including bioanalytics,^1,2^ bioreactor design,^3,4^ biosensing,^5,6^ and biocatalysis.^7,8^ The increasing interest in its application over conventional techniques can be attributed to the flexibility and freedom to fabricate complex geometries, possibility to customize the design to enhance the material’s properties, as well as material savings and production cost reduction together with high reproducibility.^9^

In biocatalysis, 3D printing has been employed in reactor design and enzyme immobilization.^7,10^ In the latter, the biocatalyst is combined with a support material, typically polymers capable of forming hydrogels often termed as “bioink”. By assembling cellular bioentities either via extrusion-based or digital light-processing (DLP)-based printing, the 3D shape of the resulting biocatalytic matrix is controlled as well as the local concentration and spatial distribution in a modular fashion.^8^ Other advantages of enzyme immobilization include enhanced catalyst turnover, simpler downstream processing reducing production costs, and increased stability toward extreme conditions (*i.e.*., organic solvents, high temperatures, and pressures).^11^

Whole-cell immobilization or encapsulation is a widely used method for the stabilization and facilitated recovery of cellular biocatalysts. A key advantage of whole-cell biocatalysis is that cells retain their endogenous cofactors, thereby eliminating the need for external cofactor supplementation and regeneration, reducing overall reaction costs.^12^ Entrapment or encapsulation in a rigid network of polymer matrices (*e.g.*, alginate, agarose, and polyacrylamide) is the most widely preferred method to immobilize whole cells. The polymer has to be porous enough to allow diffusion of the substrates and products while protecting the microorganism from harsh reaction conditions.^11–13^ Photosynthetic microorganisms, such as cyanobacteria and microalgae, have been successfully immobilized as thin films via 3D printing in a mixture of alginate and photocurable galactoglucomannan-methacrylate for the production of ethylene and a polymer precursor, ε-caprolactone.^14^ Termed as solid-state photosynthetic cell factories (SSPCFs), the immobilized biocatalysts reduce “self-shading”, commonly reported in suspension reactions.^15,16^ Light utilization is maximized in these thin films, thereby increasing production efficiency. Moreover, by utilizing solar energy and water to provide reducing equivalents in the form of NADPH and O_2_ through water splitting, these photosynthetic microorganisms were proposed as living cell factories to produce targeted chemicals and fuels.^17–19^ Another advantage of entrapping photosynthetic whole cells in the hydrogel matrix as mentioned previously is the provision of a recycling system for the regeneration of the cofactor, NADPH, required during the reaction, increasing the overall atom economy (AE) of the process, as demonstrated in suspension reactions.^20–22^

The choice of the polymer is crucial for the properties of 3D-immobilized biocatalysts. A wide range of biopolymers have been utilized to encapsulate photosynthetic microorganisms in films, including alginate-based,^14,23,24^ TEMPO-oxidized cellulose nanofibers (TCNF),^25–27^ and recently porous nanochitin.^28^ These methods can be used to effectively produce volatile compounds (*e.g.*, ethylene, hydrogen), but their application to biocatalytic reactions involving more polar substances suffers from mass transfer limitations arising from the submicron-level of pore sizes.^29^ Recently, this was addressed by “seeding” recombinant cyanobacteria in porous nanochitin with improved pore sizes, effectively transforming a nonvolatile compound under moderate stirring.^28^

Despite advancements in the polymeric matrix and immobilization techniques, material loss and fragility limit the possibility to stir or shake the reaction solution^28^ which could be ineffective in other nonvolatile compounds. Moreover, the majority of the reactions have been performed in batch, which has limited space–time yield (STY), and suffer from decreased light penetration, particularly in large photoreactors.^30,31^ Furthermore, additional precautions should be considered when handling toxic/hazardous intermediates in situ and pressure buildup, especially when hazardous gases are formed at higher pressures.^32,33^ These challenges could be mitigated through the implementation of continuous photo­(bio)­catalytic processes. Continuous flow enables process intensification owing to the relatively low dimensions of the reactors.^30^ Indeed, continuous-flow bioreactors have already been employed using photoautotrophic microorganisms for high-density cultivation coupled with oxidation,^34^ hydrogen synthesis^35^ and waste treatment.^36,37^ An illuminated coil reactor was reported by us for the ene-reduction of 2-methylmaleimide **1a** ^38^ and oxyfunctionalization of cyclohexanone to the polymer precursor, ε-caprolactone,^21^ using a suspension of recombinant cyanobacterial cells delivered using a peristaltic pump. A higher STY was reported for the coil reactor as compared to its counterpart batch reaction, and higher cell densities (3.6 g_DCW_ L^–1^) could be utilized without a significant decrease in the product formation rate.

In this work, we explored the benefits of 3D printing and continuous-flow photocatalysis by increasing the mechanical stability of SSPCFs by using a mixture of nanofibrillated cellulose (NFC) and alginate. NFC gels have a relatively high mechanical strength and can be processed into anisotropic 3D-printed structures.^39,40^ By combining NFC with alginate, the polymer mixture could be easily cross-linked by calcium ions after 3D printing without the need for photoinitiators, as reported in other studies.^14^ Photoinitiators commonly rely on ultraviolet light, which has been shown to adversely affect living cells^41^ due to the production of radicals (*e*.*g*.*,* singlet oxygen, superoxide, hydrogen peroxide),^42^ leading to reduced growth, diminished photosynthetic pigment content, and oxidative damage.^43^ Due to these concerns, photoinitiators were not used in the present study. Instead, 3D printing and cross-linking with divalent ions were performed at room temperature and under very mild conditions, which are beneficial for whole-cell encapsulation. The physiological state of the cells after 3D printing was assessed by measuring the effective yield of Photosystem II (Y­(II)) and oxygen evolution, both key indicators of photosynthetic efficiency and productivity of photoautotrophic microorganisms.^44^ The mechanical stability of the 3D-printed films was determined by tensile strength measurements to ensure structural robustness surpassing other cyanobacterial entrapment techniques used under submerged reaction conditions.

To demonstrate its applicability in whole-cell biotransformation, the NAD­(P)­H-dependent ene-reduction of 2-methylmaleimide **1a** was chosen as the model reaction mediated by recombinant cyanobacteria *Synechocystis* sp. PCC 6803 harborings the *yqjM* gene from *Bacillus subtilis*. The reaction was initially characterized in batch to determine the influence of the optical cell density on product formation rates and specific activities. Using the optimum cell density, photobioreactors were subsequently constructed, and the reaction was performed in a continuous flow setup to increase STY of the reaction. Mass transfer in the photobioreactor was enhanced by improving mixing using a tailor-made design fabricated via 3D printing for the stereoselective continuous production of (*R*)-2-methylsuccinimide **1b**. Lastly, the environmental impact of the reaction was determined by calculating pertinent sustainability parameters.

## 2 Results and Discussion

### 2.1 Bioprinting Catalyst from Biocompatible Polymers

To generate films with diverse geometries and enhanced mechanical stability, 3D printing via direct-ink-writing (DIW) was employed. The NFC/alginate blend provides a transparent^45^ and biodegradable matrix^39^ suitable for entrapping cyanobacterial cells for light-driven whole-cell biotransformations (Figure 1). Moreover, the exceptional mechanical performance of NFC—exhibiting an elastic modulus values up to approximately 140 GPa^45^—combined with its low density can contribute to the robustness of the resulting films. A bioink consisting of whole cells of *Synechocystis* sp. was combined with a mixture of NFC and alginate (ALG) and 3D-printed at room temperature via DIW into rectangular strips having dimensions of 1 cm × 3 cm (Figure S1). The three-component bioink was mixed with a self-made and 3D-printed stirrer previously reported.^39^ The stirrer design enabled a production of a homogeneous bioink directly in the printing cartridge in less than 10 min, preventing the highly viscous components from sticking to the cartridge wall, therefore avoiding material loss. The stability of the films was tested by printing either one or two layers for full films, while the effect of increased surface area on the cell physiology and biotransformation rate was determined by printing a “mesh” geometry (Figure 1, bottom). The 3D-printed films were “self-standing” (Figure 3A) and had high shape fidelity even after cross-linking using CaCl_2_ and NaCl and exposure to high agitation (<140 rpm). This allowed for an improved mass transfer of the reactants during the biotransformation. Compared to other works using 3D-printed photoautotrophic microorganisms,^14^ the 3D-printed films were not photocured, rather ionically cross-linked using a solution of CaCl_2_ and NaCl. Albeit possible after optimization, photocuring can have adverse effects on living cells due to the formation of free radicals from monomers or initiators.^41,43^ Hence, cross-linking after 3D printing offers milder reaction conditions for bioprinting and (reversible) cross-linking of cells as compared to photocuring.

**Figure 1:**
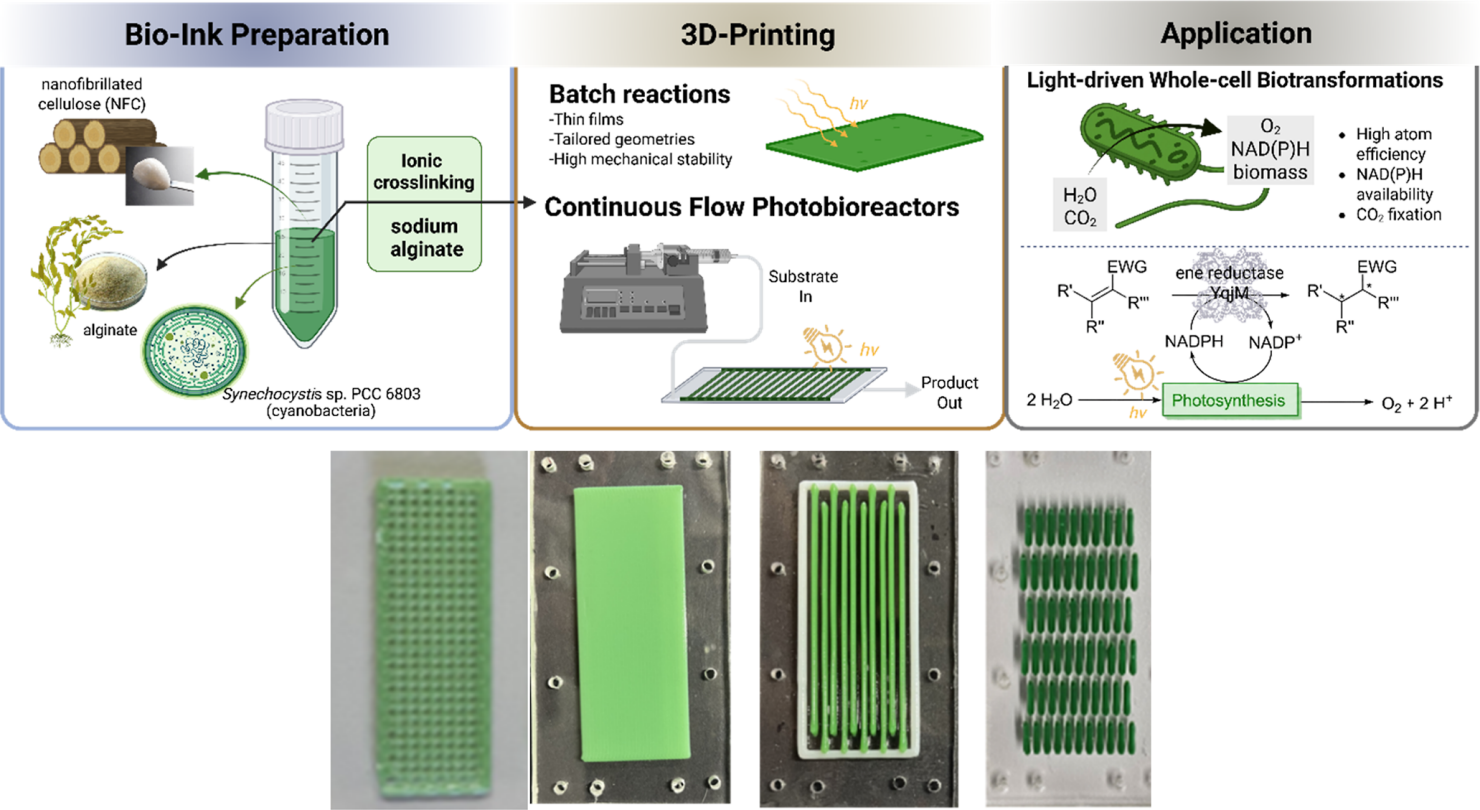
Top: Schematic diagram of 3D printing of the biocatalyst containing the three components, NFC, alginate, and cyanobacteria, to create films with various geometries (bottom), continuous photobioreactors via 3D printing (bottom: mesh (batch film), full, string, and line geometries). 3D-printed films and bioreactors with various geometries can be applied in light-driven whole-cell biotransformations such as ene-reductions driven by water oxidation to increase the atom economy of the process.

**Figure 2:**
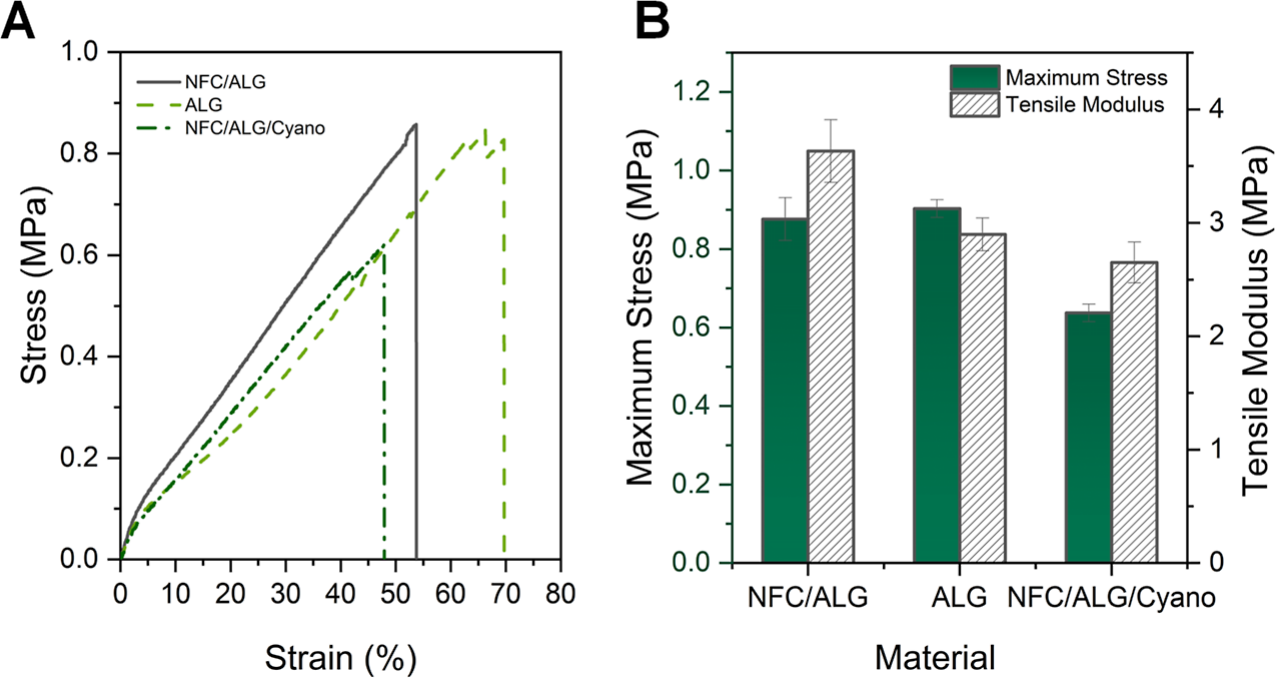
(A) Tensile stress–strain diagrams and (B) comparison of mechanical properties of the different 3D-printed biocomposites. For the composite containing cyanobacteria, the initial cell concentration was *ca.* 6–7 g_DCW_ L^–1^.

**Figure 3:**
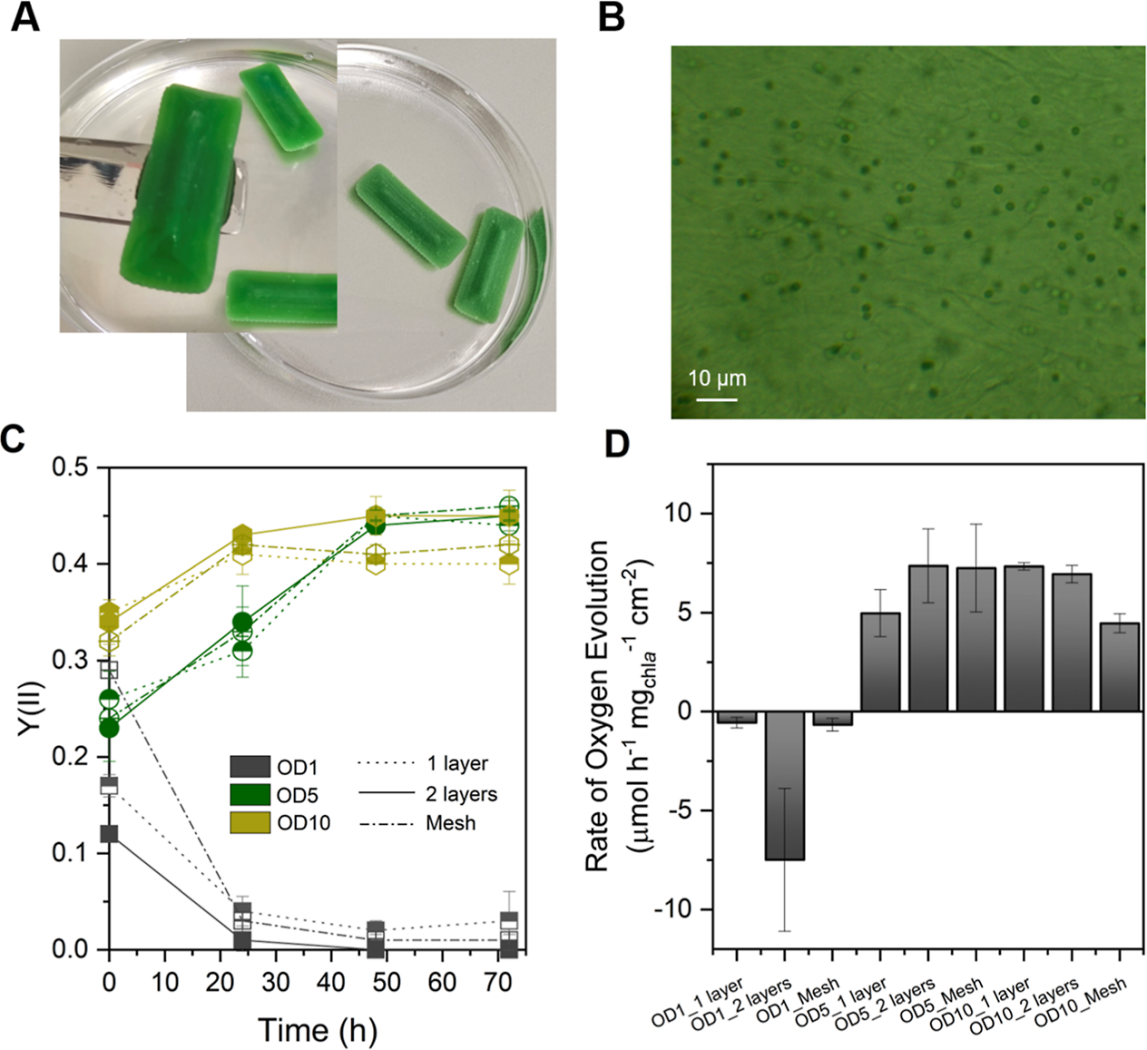
Immobilized cyanobacteria via 3D printing using the NFC/alginate composite. (A) Immobilized *Synechocystis* sp. PCC 6803 in NFC/alginate with film dimensions of 1 cm × 3 cm after cross-linking with CaCl_2_ and NaCl salts. (B) Light microscopy images of 3D-printed immobilized *Synechocystis* sp. in NFC/alginate showing cyanobacteria cells with ca. 2 μm in diameter. (C) Change of effective yield of Photosystem II in 3D-printed wild-type *Synechocystis* sp. in two geometries (full—1 or 2 layers and mesh) at various cell loadings. (D) Rate of oxygen evolution of the 3D-printed biocatalyst. OD1 corresponds to 0.24 g_DCW_ L^–1^ of *Synechocystis* sp.^16^

### 2.2 Mechanical Properties of the 3D-Printed Films

Achieving mechanical stability of photosynthetic cell factories in a film geometry remains challenging and typically requires additional physical supports, such as foam sponges^24,25^ or insect screens.^23^ Although materials with improved rheological properties compared to pure alginate-based matrices have been developed, their application has, thus far, been limited to biotransformations involving volatile compounds. In such systems, rigorous shaking is not required to mitigate mass transfer limitations. In contrast, reaction systems involving nonvolatile compounds, as investigated in this study, demand mechanically robust films capable of withstanding substantial agitation.

The films’ uniaxial tensile strength was measured using dog-bone specimens (Table S2 and Figure S2). Figure 2A,B shows the engineering stress–strain curves and tensile moduli of the 3D-printed films, respectively. All samples showed similar dynamics of film deformation with a gradual increase in stress until fracture, similar to those reported for 3D-printed NFC/ALG structures cross-linked using EtOH/H_2_O^39^ or NFC/epoxy composites.^46^ The maximum stress tolerated by NFC and ALG samples was comparable (*ca.* 0.8 MPa). However, despite showing superior toughness, the ALG samples faced critical issues with tear propagation and swelling.^47^ Immediate fracture was observed with alginate samples containing small defects such as a cut or air bubble upon application of minimal force. Without the reinforcing effect of NFC in the biocomposite, the ALG samples were prone to tear propagation, resulting in only 3 valid test results out of the 16 ALG samples tested. While ALG samples demonstrated promising mechanical properties compared to NFC/ALG, the tedious preparation (see Experimental Section) and distinct tear propagation made this biocomposite less useful as a gel matrix in a 3D-printed reactor. Furthermore, using only alginate in the mixture resulted in undesired swelling, particularly when using structures with high volume. On the other hand, the biocomposite containing cyanobacterial cells (NFC/ALG/Cyano) displayed slightly lower stress and strain at break, which could be attributed to the oxygen generated by *Synechocystis* sp. during the four-day storage period. The released oxygen may have accumulated in the printed structure, possibly disrupting its integrity and weakening the material. Furthermore, cells might competitively bind with the Ca^2+^ ions which could disrupt the interlinking between alginate and calcium ions.^26^

The measured tensile stress (max. 860 kPa) was comparable to what was reported using 3D-printed NFC/ALG biocomposites (*ca.* 360–950 kPa) designed to mimic porcine aortae for presurgical planning.^40^ Albeit having the lowest measured tensile stress (620 kPa) in this study, NFC/ALG/Cyano outperformed NFC/ALG/Gelatin hydrogels (320 kPa) reported by Han et al.^48^

The mechanical properties of the NFC/ALG/Cyano biocomposite could be attributed to the shear stress associated during printing, which allowed the fibers to align in the printing direction.^39^ Overall, the 3D-printed biocomposite matrix showed immense potential as a suitable framework to entrap photoautotrophic microorganisms exhibiting high mechanical properties, which could be beneficial in certain reactions requiring vigorous stirring to alleviate mass transfer limitations.

### 2.3 Physiological Condition of the Cells after Immobilization

After immobilization, the physiological condition of the cells was assessed by measuring the yield of Photosystem II (Y­(II)) and, consequently, the rate of oxygen evolution. Figure 3A shows the 3D-printed cells after cross-linking, which are “self-standing”, while Figure 3B shows a light microscopy image of *Synechocystis* sp. immobilized in the NFC/ALG composite. Cyanobacterial cells approximately 2 μm in diameter can be observed in the films, which is in accordance with previous measurements using electron microscopy.^49,50^ By employing a two-layer 3D-printing approach, cells incorporated within the initial layer become embedded within the interior of the construct, whereas cells deposited in the subsequent layer remain more exposed. This can be seen from the light microscopy image showing cells that seem darker (first layer) over slightly pale ones (second layer).

Figure 3C,D shows the Y­(II) yield and the rate of oxygen evolution, respectively, for wild-type *Synechocystis* sp. immobilized in NFC/ALG at various optical densities and geometries. The Y­(II) yield represents the proportion of photons of incident light utilized to drive photochemistry and is an indicator of photosynthetic activity^44^ and, hence, the state of the cells. As previously reported, cyanobacterial strains typically have a Y­(II) value around 0.4. In Figure 3C, films with lower cell loading (0.24 and 1.2 g_DCW_ L^–1^ corresponding to OD1 and OD5, respectively), regardless of the geometry, showed low Y­(II) values (<0.4) after entrapment, indicating printing-induced cellular stress. On the other hand, a higher cell loading of 2.4 g_DCW_ L^–1^ (OD10) showed a relatively higher initial Y­(II) value. The films were then incubated under low light (60 μmol of photons m^–2^ s^–1^) without agitation to help the cells recover. After 2 days, the cells with OD > 5 showed an increase in Y­(II) value (0.40), which was maintained after 3 days. Because Y­(II) values are highly dependent on cultivation conditions (*e.g.*, light intensity, CO_2_ availability, mineral supplementation, etc.) as well as the measurement protocol, direct comparison of absolute values is difficult. Nonetheless, Figure 3C shows that, with continued incubation, films with higher cell loadings (>OD1) exhibited a marked increase in Y­(II) from 0.2 to 0.45, indicating substantial recovery of photosynthetic activity.^51–53^

Furthermore, the condition of PSII was evaluated by measuring the rate of oxygen evolution at a light intensity of 250 μmol of photons m^–2^ s^–1^. The rate of oxygen release was calculated from the onset (200 s, Figure S3 and Table S3) until 10 min to have a similar duration for all the tested cell densities and geometries. As illustrated in Figure 3D, increasing the cell loading (OD 5 and 10) resulted in matrices that generated oxygen with an average of 6.38 μmol O_2_ h^–1^ mg_Chla_^–1^ cm^–2^, regardless of geometry. On the other hand, all 3D-printed films prepared using a low cell loading of 0.24 g_DCW_ L^–1^ exhibited negative O_2_ production (*i.e.*, consumption) indicative of stress. This trend aligns with the reduced Y­(II) values observed in films printed with a low cell loading.

### 2.4 Biocatalytic Reduction Mediated by 3D-Printed Recombinant *Synechocystis*

#### 2.4.1 Biotransformation in Batch Mode

To assess the applicability of the 3D-printed NFC/ALG/Cyano biofilms in stereoselective biotransformation reactions, the recombinant *Synechocystis* sp. harboring the ene-reductase from *B. subtilis* (SynP_*cpc*_::YqjM) was used. Its performance has been compared in various suspension reactions, either in batch^15,16,54,55^ or in flow.^38^ Figure 4A shows the YqjM-mediated reduction of **1a** to (*R*)-**1b** in recombinant *Synechocystis* sp. utilizing only water to provide electrons to regenerate the cofactor NADPH required for the reaction. The biotransformation was initially performed in batch with cells entrapped in 1 cm × 3 cm films that are immersed in the substrate solution, illuminated with 90 μmol photons m^–2^ s^–1^. The reaction vials were agitated at 140 rpm at 30 °C to facilitate efficient mass transfer in the films. Figure 4B shows the progress of **1b** formation over a 24 h time course using full films produced with one layer (0.5 mm) and two layers (1.0 mm), including a mesh geometry produced using two layers. Cell suspensions with a minimum cell density of *ca.* 1 g_DCW_ L^–1^ were immobilized in the matrix, as this concentration was determined to be less sensitive to stress than cells in films printed with an initial cell density of 0.24 g_DCW_ L^–1^ (Figure 3C,D). In all geometries tested, a starting cell density of 2.4 g_DCW_ L^–1^ (OD10) showed the highest product formation after immobilization (Figure 4B), with a maximum product formation of 69% after 6 h. This corresponds to a chlorophyll content of 15 μg of chl*a* per film (3 cm^2^). The volumetric productivity increased concurrently with the cell loadings, with full films showing the highest rates in all geometries tested (Figure 4C). However, when the activities were normalized to the amount of chlorophyll content, there was no remarkable difference in all cell density loadings as well as geometries (Figure 4D), with cells showing an average activity of 7–8 μmol min^–1^ (U) mg chl*a* ^–1^ for the formation of **1b**. There was no significant effect of the film geometry on the product formation rate. Interestingly, the observed activities in the films are almost 3-fold higher than suspension reactions (3 U mg_chl*a*_^–1^)^16^ using the same strain. These are indications that the cells within the thin films are not light-limited at the investigated cell densities. This is a striking result considering that the so-called “self-shading” of the cells in cell suspensions hinders light transmittance and reduces the activity of the photosynthesis-driven biotransformation at cell densities higher than OD 1–2. In comparisons between suspension and immobilized reaction systems, mass transfer limitations, especially those arising from restricted diffusion of substrates and products within the matrix, represent one of the main causes of the observed differences in performance.^56^ In photobiocatalytic processes, light availability often constitutes an additional major challenge, especially in large-scale reactors.^31^ Nevertheless, our findings indicate that, under immobilized batch conditions with sufficient agitation, cell concentration plays a significant role in enhancing product formation rates, and increasing it until an OD of 10 (2.4 g_DCW_ L^–1^) does not adversely affect the specific activity.

**Figure 4:**
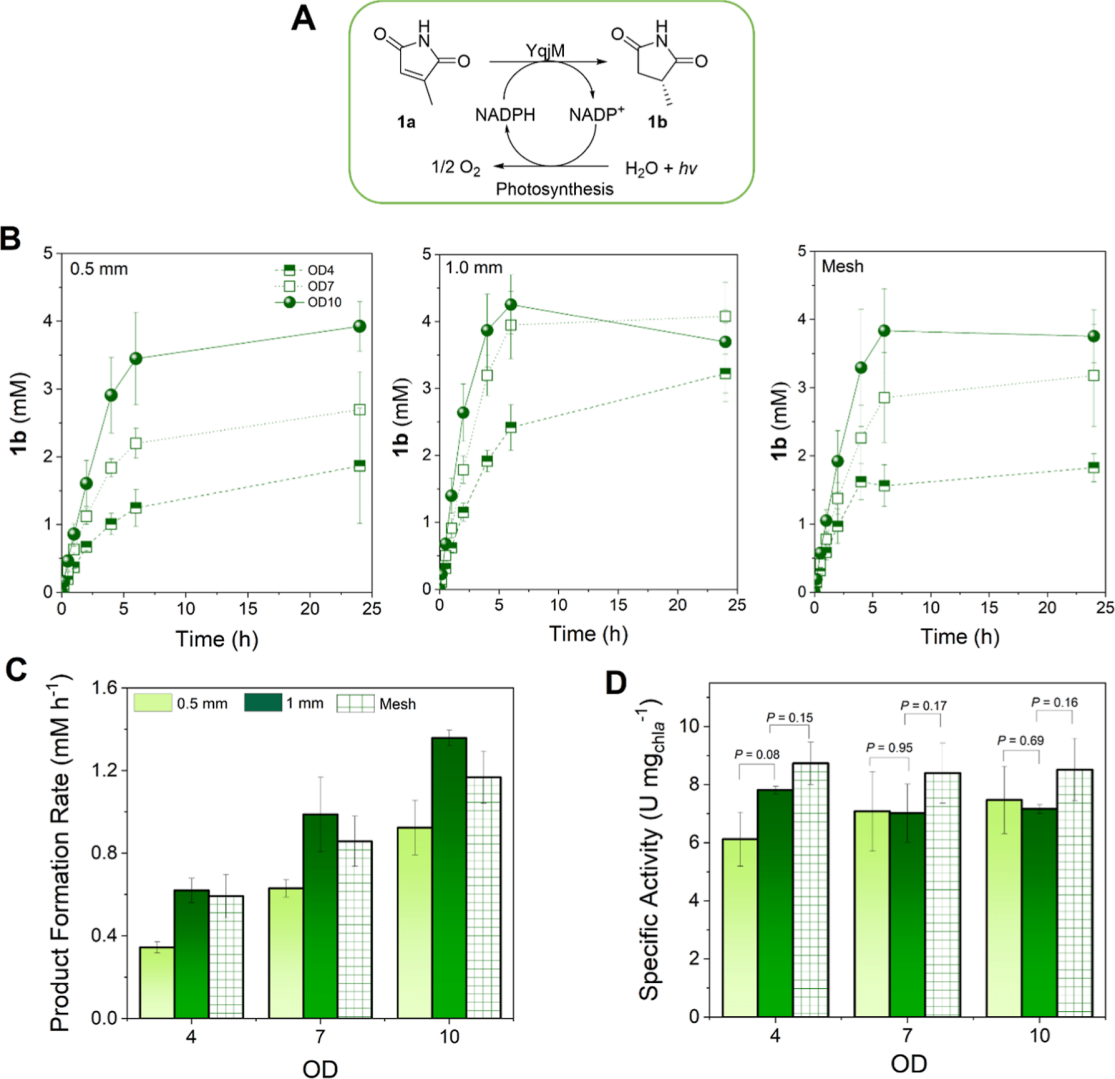
Whole-cell biotransformation of **1a** mediated by 3D-printed recombinant *Synechocystis* sp. expressing the *yqjM* gene from *B. subtilis*. (A) Light-driven reduction of **1a** by ene-reductase YqjM using photosynthesis for cofactor regeneration mediated by recombinant *Synechocystis* sp. (B) Time profile of **1b** formation in 3D-printed Syn::P_*cpc*_YqjM (1 cm × 3 cm) at OD_750_ = 4, 7, 10 (corresponding to 0.96, 1.7, and 2.4 g_DCW_ L^–1^, respectively) using three different film geometries over 24 h operation time. (C) Initial product formation rates in the formation of **1b** at different optical densities and film geometries and (D) specific activities normalized to chlorophyll *a* (chl*a*) content. The three tested geometries were full films with thicknesses of 0.5 mm (light green) and 1 mm (dark green) and mesh films (thickness of 1 mm) using optical densities of OD4, 7, and 10. The reaction was supplemented with CaCl_2_ (5 mM) to retain the structural integrity of the films throughout the biotransformation. *Reaction conditions:* [*C*_0_] = 5 mM, 5 mL, 30 °C, light intensity of 100 μmol photons m^–2^ s^–1^, 140 rpm, *N* = 3. *P* values were calculated using Welch’s *t* test.

To demonstrate the robustness of our proposed immobilization technique, we also entrapped recombinant *Synechocystis* in thin alginate films. Entrapping photoautotrophic cells in alginate has been studied as an immobilization technique, particularly in the production of volatile compounds such as hydrogen^23,57^ and ethylene.^24^ These films required a support material such as window screens^23^ or melamine foam sponges^24,25^ and were not agitated during the reaction. For nonvolatile compounds such as **1a** and **1b**, mass transfer of the compounds through the matrix requires shaking. In fact, when reactions were performed without agitation, we observed 2-fold lower product formation rates (Figure S4). To increase the mass transfer, the reactions were agitated at 140 rpm for 24 h. Albeit having a mechanical support, alginate thin films could not tolerate the high stirring and showed signs of deterioration after 4 h. In contrast, the 3D-printed films maintained their rigidity and fidelity after 24 h of reaction at a shaking speed of 140 rpm (Figure S5). Their higher mechanical strength is thus an important advantage for biotechnological processes involving mass transfer limitations.

The high mechanical stability of 3D-printed films could be attributed to the high aspect ratio of NFC, which, when combined with the shear forces generated as the ink is extruded through the narrow nozzle during printing, helps align the fibers in the direction of printing. This was also corroborated by the tensile strength measurements shown in Figure 2. It was previously shown that fibers aligned uniaxially has 2-fold higher modulus compared to those aligned perpendicularly.^39,58^ Hence, by entrapping cyanobacterial cells in NFC/ALG and 3D printing, we were able to improve the product formation rates by increasing the mechanical rigidity of the films. This expands the reaction system to mass transfer demanding applications including phase transfer or high-molecular-weight reactants or products.

#### 2.4.2 Biotransformations within the 3D-Printed Bioreactor Systems

The 3D-printed system was then translated to a continuous photobioreactor with the aim of increasing the surface area, tuning the flow geometries, and scaling the process to ultimately enhance the overall space–time yield (STY) and downstream processing.^59^ The low film thickness (1 mm) enables efficient light penetration even at higher cell loadings^31,38^ due to the improved surface area. Using the optimum cell density of 2.4 g_DCW_ L^–1^ (OD10) obtained from batch experiments, continuous photobioreactors with varying geometries were constructed with NFC/ALG (i.e., full, string, and line, Figure 5A) with the aim of improving mixing by creating interstitial volumes and thereby increasing turbulence. Surface coating of the polycarbonate plexiglass was performed to fix the bioink to the reactor’s surface, seal it against bottom leakage, and control swelling during cross-linking (Figure S6). This approach was time-efficient (i.e., average of 4 min per bottom plate) to ensure a flat surface with reduced ink consumption as compared to creating an anchor profile. After UV–ozone treatment and coating with polyethylene imine (PEI, 5% w/v), the bioink was deposited on the surface and further cross-linked with CaCl_2_ (75 mM) and NaCl (100 mM). This concentration of salts also showed the least amount of swelling in the photobioreactor. Lastly, the reactor was sealed using a tailored silicone sealing (Sylgard 184) which is known to be biocompatible.^60^

**Figure 5:**
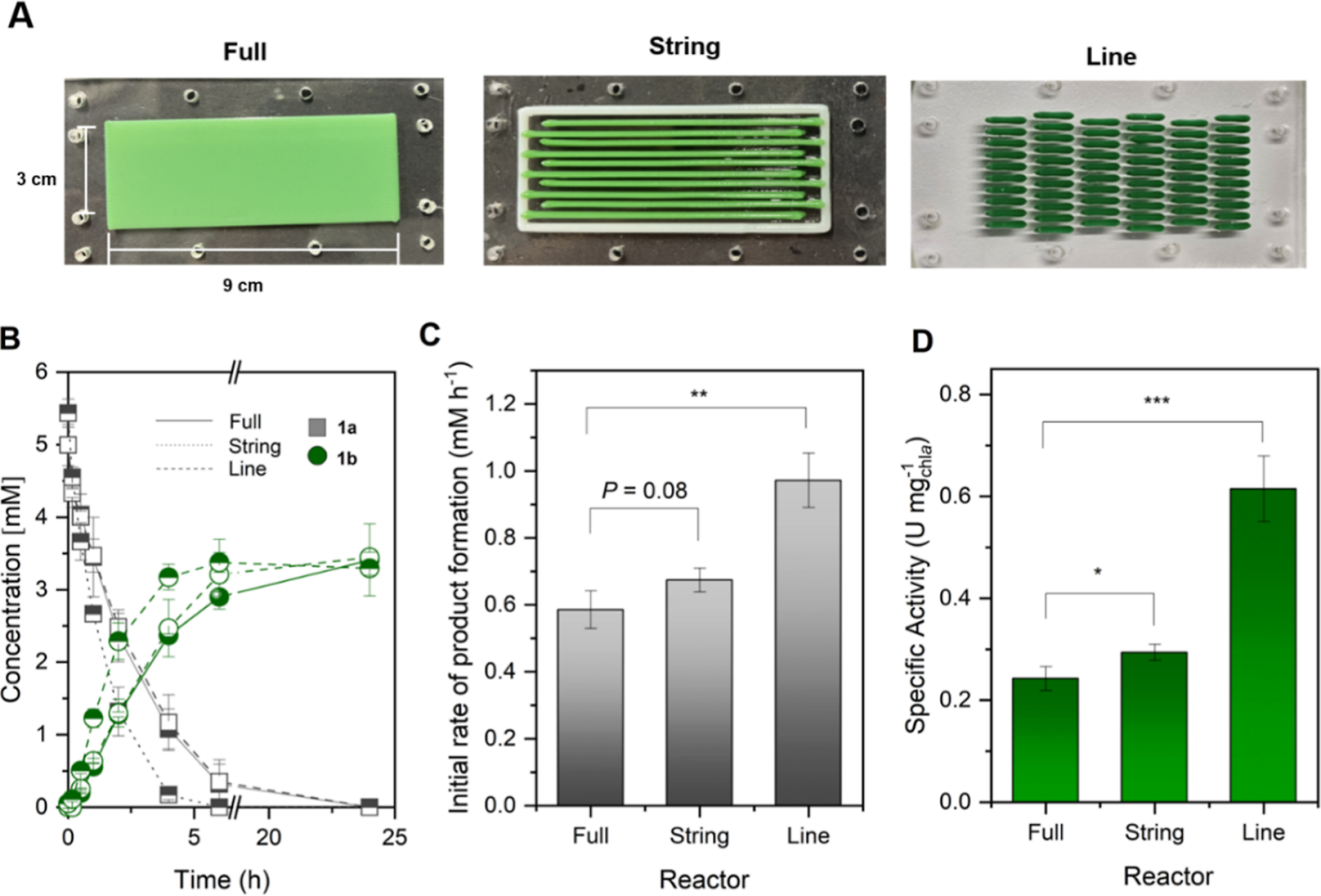
YqjM-mediated biotransformation of 1a in recombinant *Synechocystis* sp. immobilized via 3D printing in a recycle mode bioreactor design comparing three different geometries: full, string, and line. (A) Three recycle mode bioreactors utilized in this study for recycle studies having various geometries. (B) Time course of substrate consumption and product formation up to 24 h operation time. (C) Initial rate of **1b** formation (initial rate was calculated with the product concentration less than or equal to 10%) and (D) whole-cell-specific activities normalized to the chl*a* content at a flow rate of 0.8 mL min^–1^. The reaction was also supplemented with CaCl_2_ (5 mM) to retain the structural integrity of the films throughout the biotransformation. *Reaction conditions:* [*C*_0_] = 5 mM in BG-11, 0.8 mL min^–1^, RT, 2.4 g_DCW_ L^–1^ (OD10), light intensity of 100 μmol photons m^–2^ s^–1^, *N* = 3.

Using the same reaction as in batch mode, a continuous flow process was designed by placing the reactors under a light source delivering *ca.* 90 μmol of photons m^–2^ s^–1^ (Figure S7). The substrate solution (5 mM **1a**) supplemented with CaCl_2_ (5 mM) immersed in a water bath set at 30 °C was then delivered to the reactor by a peristaltic pump operated at 0.8 mL min^–1^ in recycle mode. By performing the biotransformation in recycle mode, conversion efficiency is improved, potential substrate inhibition is alleviated, and the effective contact time between the substrate and the immobilized biocatalyst is increased.^61–63^ Figure 5A shows the three types of photobioreactors utilized in this work having overall dimensions of 9 cm× 3 cm and a total thickness of *ca.* 1 cm. Table 1 shows several pertinent parameters to characterize the reactors. Figure 5C shows that the line reactor has the highest product formation rate (1 mM h^–1^) among the tested reactors, achieving 58% product yield after 4 h. On the other hand, both the full and string reactors required 6 h to achieve the same level of product yield. Normalizing the rates to the amount of cells (measured using chlorophyll content determination) further affirmed the superior activity of the line reactor (Figure 5D).

**Table 1:** Characterization of the Recycle Mode Bioreactors Utilized in the Biotransformation of **1a** Using 3D-Printed Recombinant *Synechocystis* sp. in This Study^a^

	reactors		
parameters	full (*N* = 6)	string (*N* = 8)	line (*N* = 3)
mass of bioink (g)	4.06 ± 0.56	2.16 ± 0.35	3.23 ± 0.21
height of bioink (mm)	1.24 ± 0.05	1.50 ± 0.13	3.50 ± 0.05
specific surface area (mm^2^ g^–1^)^b^	625.30	1017.69	2129.74
active solution volume (cm^3^)^c^	4.09 ± 0.41	2.23 ± 0.16	5.18 ± 0.81
residence time (min)^d^	7.13	9.33	15.02
flow velocity (cm min^–1^)^e^	1.12	38.1	2.77

a *N* corresponds to the number of replicates per reactor.

b Calculated using Autodesk Inventor by taking a photo of the bioreactor after cross-linking.

c Active solution volume was defined as the amount of solution directly in contact with the 3D-printed biocatalyst at a given time.

d Calculated at a flow rate of 0.8 mL min^–1^ based on conductivity measurements (see Supporting Information).

e Calculated at a flow rate of 0.8 mL min^–1^ multiplied by the cross-sectional area.

The higher activity in the string reactor compared to the full reactor (Figure 5D) could be attributed to its higher specific surface area (1017.69 mm^2^ g^–1^) and flow velocity (38.1 cm min^–1^) (Table 1). This suggests enhanced mixing alleviating mass transfer limitations in the reactor. While higher flow rates can improve mixing by reducing mass transfer limitations, they did not significantly influence the relative performance of the string and line reactors in this study. Rather, differences in mixing arose from the reactor geometries themselves. In the string reactor, the flow is diverted only around each string, but in the line reactor, it is repeatedly split and merged, generating substantially better mixing.

To obtain more insights into the flow behavior inside the reactors, residence time distribution (RTD) experiments were carried out. Initial runs to determine the residence time using methylene blue as the UV tracer were unreliable due to its adsorption by the hydrogel. Thus, the tracer was changed to an electrolyte solution, which was analyzed with a conductivity sensor. For this setup (Figure S8), a flow cell with a low internal volume was 3D-printed to enhance measurement precision. The line reactor showed the highest specific activity which could be ascribed to its higher surface area (2129.74 mm^2^ g^–1^) and the longer residence time of the solution in the reactor (15 min) compared to the full (7 min) and string reactors (9 min); see Table 1. The high specific surface area improved the interaction of the immobilized recombinant cyanobacteria with the substrate solution, resulting in improved catalytic performance and overall activity.^64^

Moreover, compared to the string reactor, the line reactor displayed a broader RTD curve and lower Bodenstein number (*Bo*) (Table S4 and Figure S9), indicative of enhanced back and axial mixing.^65^ This deviation from plug flow aligns with theoretical predictions, where reactors with lower *Bo* numbers exhibit broader RTD and enhanced back-mixing.^63^

Based on the bioreactor results, a continuous line reactor was 3D-printed to deliver the substrate on one end of the reactor while recovering the product on the other side. A larger reactor (17.5 cm × 6 cm, 2-fold larger than that in Figure 5A) was produced. Figure 6 shows the bioreactor setup and progress of the reaction. A higher substrate concentration (10 mM) and a lower flow rate (0.1 mL min^–1^) were utilized to increase the STY and the residence time, respectively, of the reaction. After 4 h, a steady-state **1b** concentration of 6.5 mM was observed, which was maintained even after 24 h. The mass balance was noticeably incomplete, with only *ca.* 60% of the substrate converted to the product. Hence, the immobilized catalyst was scraped off the plexiglass support after the reaction, extracted with ethyl acetate, and subjected to gas chromatographic analysis. Figure S10 shows the chromatogram of the extracts showing mainly the product **1b**. The concentration was calculated to be 4.7 mM, closing the mass balance. Additionally, a “blank” line reactor was constructed to study the adsorption of either **1a** or **1b** in the polymer matrix. Figure S11 shows that the polymeric matrix adsorbs 2-fold higher **1a** as compared to **1b**. The affinity of either the substrate or the product to a polymeric material (*i.e.*, nanochitin) was also confirmed using surface plasmon resonance spectroscopy (SPR),^28^ which showed a 5-fold higher affinity of **1a** compared to **1b**. This indicates that both the polymer matrix and the cells adsorb the compounds.

**Figure 6:**
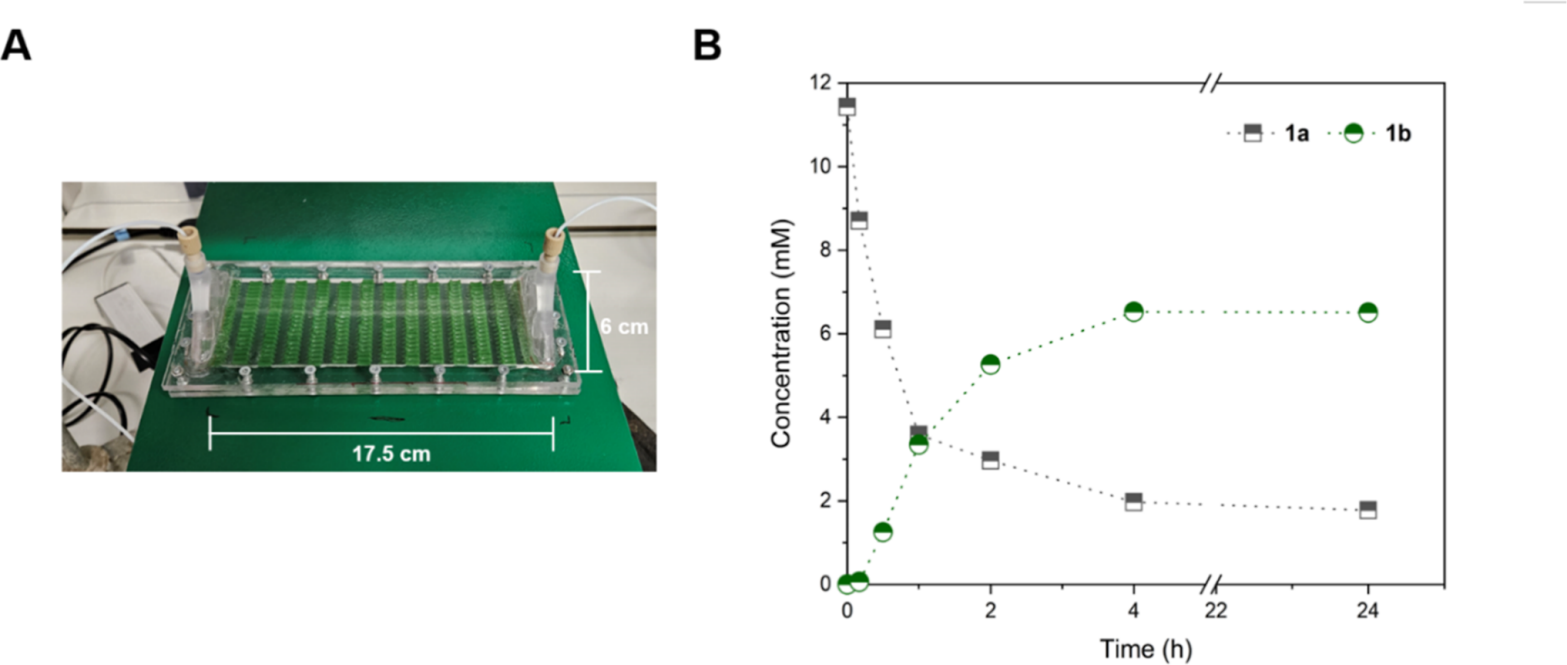
Continuous biotransformation of **1a** mediated by 3D-printed recombinant *Synechocystis* harboring YqjM. (A) Reaction setup consisting of a 3D-printed bioink having dimensions of 17.5 cm × 6 cm and (B) progress of the reaction catalyzed by recombinant *Synechocystis* immobilized within 3D-printed NFC/ALG films monitored for 24 h operation time, which reached steady state at 6.5 mM **1b** concentration. The substrate solution was delivered to the reactor using a syringe pump with a residence time of 30 min. The reaction was supplemented with CaCl_2_ (5 mM) to retain the structural integrity of the films throughout the biotransformation. *Reaction conditions:* [*C*_0_] = 10 mM, 0.1 mL min^–1^, 30 °C, 2.4 g_DCW_ L^–1^ (OD10), light intensity of 100 μmol photons m^–2^ s^–1^, *N* = 1.

Nevertheless, an STY of 0.28 g L^–1^ h^–1^ was calculated for the continuous reactor. Furthermore, *R*-**1b** was obtained with an enantiomeric excess (ee) of >99% using the line reactor (Figure S12), similar to other reactor types which utilized recombinant *Synechocystis* sp. expressing the *yqjM* gene.^15,38^

### 2.5 Sustainability Assessment of the 3D-Printed Biocatalytic Reactors

As illustrated in Figure 1, light-driven biotransformations fueled by water oxidation exhibit high atom efficiency due to the availability of reducing equivalents in the form of NAD­(P)­H. This inherent supply of cofactors is particularly advantageous for NAD­(P)­H-dependent redox reactions, eliminating the usage of sacrificial cosubstrates for cofactor regeneration. The sustainability of the 3D-printed reactor concept utilizing recombinant cyanobacteria was assessed by calculating several parameters related to AE, such as the relative mass economy (RME) and the optimum efficiency (OE), as described by McElroy et al.^66^ The use of photoautotrophic microorganisms as production hosts has been often emphasized as a sustainable process due to their minimal growth requirements and their ability to fix carbon dioxide and regenerate cofactors in the form of NADPH. The latter is crucial in cofactor-dependent reactions eliminating the need for a recycling system to render the biotransformation economically feasible, which results in decreased AE of the process.^20–22^ The AE of a reaction defines the number of atoms of the reactants appearing in the product.^66,67^ Using whole cells of heterotrophic *E. coli* harboring ene-reductases, the cofactors are often regenerated by either supplementation of sacrificial cosubstrates such as glucose^68^ or formate^69^ or coupling the reaction with another enzyme system. Both routes can potentially reduce the AE with the reported 78% and 49% when formate^69^ and glucose^68^ were utilized as cosubstrates for cofactor regeneration, respectively. The lower AE observed with glucose addition can be attributed to its relatively higher molecular weight, particularly when excess glucose is added to compensate for metabolic losses.

However, when recombinant *Synechocystis* sp. harboring ene-reductases are utilized particularly in the reduction of **1a**, an AE of 88% was reported^20,70^ by utilizing only water as an electron donor to regenerate NADPH in the photosynthetic electron transport chain. To gain deeper insight into the reaction efficiency, we have calculated pertinent green sustainability metrics for **1a** reduction mediated by recombinant *Synechocystis* sp. harboring the ene-reductase YqjM in various reactor concepts (Table 2). The comparison was performed at an operating time of 4 h for all the reactors. Hence, some of the parameters are calculated based on published works. From Table 2, it is observed that the line reactor outperformed all the other photobioreactor geometries (*i.e.*, full and string) in terms of RME (78%) and OE (91%), which could be attributed to the higher specific surface area and possibly longer retention time of the liquid in the bioreactor (Table 1). This is at par with values obtained using the coil reactor, suggesting that the increased surface-area-to-volume (SA–V) ratio played a major role in both reactors in terms of reaction efficiency.

**Table 2:** Green Chemistry Parameters for the Reduction of **1a** Mediated by Recombinant *Synechocystis* sp. Harboring the YqjM Ene-Reductase Performed in Various Reactor Concepts at 4 h

	this study				other works		
reactor	full	string	line	continuous	flat panel	coil	BCR
RME^a^,%	48.3	50.2	78.0	65.1	62.0	77.9	37.0
OE^b^,%	55.1	57.3	90.8	74.3	70.8	89.0	42.2
sEF^c^	12.8	11.7	7.2	7.0	2.5	2.5	5.7
cEF^d^ (× 10^3^)	3.7	3.6	2.3	1.4	0.3	0.3	0.6

a Calculated as the mass of product over the total mass of reactants.

b OE = RME/AE.

c Refers to the simple E-factor, excluding water from the calculation.

d Complete E-factor including water. Details can be found in Table S5 in the Supporting Information.

Furthermore, the sustainability of the process was evaluated by determining the E-factor, which is defined as the total amount of waste generated over the amount of product. For this parameter, the simple E-factor (sEF) is recommended for lab-scale processes or early surveying of plausible reaction systems.^67^ Among the immobilized bioreactors, the line reactor and its corresponding continuous mode showed the lowest sEF (*ca.* 7). However, this value is higher compared to other reactor concepts reported for a similar reaction (sEF = 2–6, Table 2). This can be attributed to a 10-fold lower substrate concentration (5–10 mM) fed in the immobilized reactors compared to other works (40–50 mM) which utilized a fed-batch approach. Although not recommended for small-scale reactions, the cEF was also calculated for the immobilized bioreactors. From Table 2, it can be seen that using the continuous reactor, the cEF is 2–5 times higher as compared to previous studies, hinting on the significant contribution of wastewater. However, we envision that by immobilizing cyanobacterial whole cells and running the bioreactors continuously for a longer time, the contribution of cell cultivation could further decrease the E-factor. As previously shown, cultivation accounts for 77–88% of the E-factor.^20^ Another route to increase the EF is to similarly perform a fed-batch approach to increase the substrate concentration or perform several recycling runs.

The space–time yield (STY) achieved in this study (280 mg L^–1^ h^–1^) is comparable to values reported for other photobiotransformations performed in various photobioreactor configurations (65–226 mg L^–1^ h^–1^).^15,38,71,72^ The turnover number (expressed as g product g^–1^ cells) for the line reactor was calculated to be 0.42, which is lower than those reported for the coil reactor (1.2)^38^ and the bubble column reactor (0.92).^15^ This difference can largely be attributed to the substantially lower initial substrate concentration used in the present study—at least 4-fold lower than that in the aforementioned systems—suggesting that higher substrate loadings would proportionally increase the TON. Beyond STY, the product yield was also evaluated and reached 0.67 g product g^–1^ substrate. This yield is in good agreement with those reported for other upscaled photobioreactor concepts (0.88–1.0).^15,20,54,71,72^

Lastly, the carbon footprint of the process was evaluated by calculating the global warming potential (GWP), expressed as kg CO_2_ per kg product.^73^ Since the reaction was performed in aqueous medium, the GWP was calculated to be 3.09 kg of CO_2_ per kg of product (equations in the Supporting Information). However, if a downstream processing with ethyl acetate extraction was considered, an additional 200 kg of CO_2_ per kg of product was calculated, underscoring the substantial impact of wastewater treatment in aqueous-based biotransformations. These findings highlight the importance of increasing product titers—*e*.*g*.*,* by employing higher initial substrate concentrations—to reduce the overall environmental footprint.

The combination of 3D printing with material science, whole-cell photo­(bio)­catalysis, and reaction engineering opens numerous innovative opportunities for new and more efficient chemical processes. High value-added products can be sustainably produced, reducing wastes and energy consumption.^10^ This work diversifies the application of nanocellulose alginate gels often used for biomedical purposes^40,74^ to biotechnological processes, particularly by integrating enzyme and cell immobilization and continuous photo­(bio)­reactor construction.

## 3 Conclusions

In this work, the applicability of 3D printing of biobased hydrogels was expanded beyond enzyme immobilization to photobioreactor design for continuous stereoselective chemical production. This was demonstrated through extrusion-based 3D printing of a bioink consisting of recombinant cyanobacteria producing an ene-reductase and biocompatible polymers such as sodium alginate and NFC. Direct-ink-writing of the bioink enabled fabrication of films with exceptional mechanical stability facilitating efficient mass transfer through agitation during the biotransformation of a nonvolatile compound. Viability studies showed that the encapsulated cyanobacteria are still capable of evolving oxygen and show high effective yield of PSII. Light limitation among the tested film thicknesses and geometries was not observed during the batch reaction, and the product formation rate was mainly influenced by the cell loading. In continuous-flow biocatalysis, the design of the 3D-printed photobioreactor played a major role, with the line reactor demonstrating the highest rate of product formation. This was attributed to a broader RTD and lower Bodenstein number in the line reactor as compared with the string reactor, allowing increased back-mixing. The main advantages of the NFC/alginate blends are their mechanical strength supporting adhesion, tear resistance, high porosity, water content dimensional stability, and the mild conditions of the ionic cross-linking compared to methods of photocuring. The 3D printing of the material allows the design of continuous-flow photobioreactors with optimal light availability and mass transfer across the liquid–solid interface. The sustainability assessment indicated that increasing the substrate concentration can potentially reduce waste formation and consequently lower the E-factor. Although this study presents a conceptual design for an immobilized whole-cell continuous bioreactor, operating it for extended periods could substantially decrease wastewater generation, primarily by minimizing the impact of cell cultivation. This study also initiates the need for further improvements of the polymer matrices or the design of effective *in situ* product extraction techniques to alleviate the adhesion of the compounds to the matrix. The environmentally benign approaches presented here could open new frontiers for various enzymatic whole-cell reactions and cell cultures under heterogeneous conditions, particularly those requiring cofactors and oxygen.

## 4 Experimental Section

### 4.1 Chemicals

The NFC suspension (Sappi Valida, 3 wt % solid content) was kindly donated by Sappi (Maastricht, The Netherlands). Alginic acid sodium salt from brown algae, calcium chloride, polyethylene imine, and *n*-decanol were purchased from Sigma-Aldrich (St. Louis, USA). Sodium chloride and ethyl acetate were purchased from VWR Chemicals (Vienna, Austria). The compound 2-methylmaleimide **1a** was synthesized as previously described.^70^ SylgardTM 184 silicone Elastomer Kit was purchased from Dow (California, USA). Polyethylene terephthalate glycol (PETG) black filament was obtained from 3D Jake (Paldau, Austria). The highly clear resin was purchased from Anycubic (Shenzhen, China).

### 4.2 Strains and Culture Conditions

*Synechocystis* sp. harboring the ene-reductase YqjM from *B. subtilis* under the control of the *cpc* promoter (P_*cpc*_)^15,16,38,54,55^ was cultivated in a plant growth chamber maintained at 30 °C and constantly illuminated, with white fluorescent lamps at an average intensity of 100 μmol photons m^–2^ s^–1^. Strains were stored in glycerol stocks (10% v/v) and reactivated by streaking out in agar plates (1.5% w/v) containing the appropriate antibiotic. Seed cultures were grown in a BG-11 liquid medium under ambient carbon dioxide. A working volume of 100 mL was prepared in a 300 mL Erlenmeyer flask and placed on top of a rotary stirrer agitated at 140 rpm. Kanamycin (50 μg mL^–1^) was supplemented to maintain the YqjM integration cassette. Exponential growth phase was reached after 4–5 days (optical density at 750, OD_750_ = 1–2) after which the cells were harvested and concentrated by centrifugation (15 min, 24 °C, 3220 *g*).

### 4.3 Bioink Preparation and 3D Printing

#### 4.3.1 Polymer Composite and Bioink Preparation

The ink was prepared by mixing NFC and alginate (15:1 w/w) in a falcon tube at 2000 rpm for 10 min using an in-house 3D-printed stirrer.^39^ Concentrated NFC with 4.5% (w/w) solid content was prepared by vacuum filtration of the 3% (w/w) NFC. Subsequently, NFC containing 3% and 4.5% (w/w) solid content was utilized to prepare the bioink with an OD_750_ = 4 and OD_750_ = 10, respectively. NFC containing 3% (w/w) solid content was filtered to a final solid content of 4.5% (w/w) using vacuum filtration. The bioink was prepared by combining the prepared polymer composite with cyanobacterial cells to a final OD_750_ = 4 or OD_750_ = 10 and was utilized immediately for 3D printing.

#### 4.3.2 Dimensional Printing, Cross-Linking, and Storage

The bioink was extruded from a polyethylene-based plastic barrel fitted with tapered tips (Nordson, UK) having an inner diameter of 0.41 mm and an extrusion pressure of *ca.* 60 kPa. In control reactions without cyanobacteria, the pressure was increased to *ca.* 115 kPa. All inks were 3D-printed using BioScaffolder 3.2 software (GeSiM, Germany). For batch reactions, two types of films (*i.e.*, full and mesh) having dimensions of 1.1 cm × 3.2 cm were 3D-printed (Figure S1). Full print matrices were produced by using two film thicknesses (0.5 and 1 mm) to determine the effect of cell loading. After 3D printing, the films were then immersed for 5 min in a solution of CaCl_2_ (50 mM) and NaCl (75 mM) for cross-linking, transferred to a fresh BG-11 medium overnight, and incubated at a light intensity of 50 μmol photons m^–2^ s^–1^ under light stirring (60 rpm).

The bioreactors for continuous flow were made of transparent RS Pro polycarbonate plastic plates (Gmünd, Austria), with inlets and outlets drilled on the top plate (additional information can be found in the Supporting Information) measuring ca. 90 mm × 30 mm. The surface of the bottom plate was treated with ozone (PSD Pro Series Digital UV Ozone System, USA) for 15 min. Three reactor designs were fabricated to increase the contact area with the substrate (Figures S6 and 5). Afterward, the printing surface was hydrophilized and treated with 5% w/v PEI solution for 2 min, followed by rinsing with ddH_2_O. The bioreactor geometries were submerged into the cross-linking solution consisting of 100 mM CaCl_2_ and 75 mM NaCl for 30 min. Similar to films, the bioreactors were allowed to soak in BG-11 containing 5 mM CaCl_2_ overnight prior to biotransformation. Additional information on the fabrication of the reactor can be found in the Supporting Information.

### 4.4 Characterization of the 3D-Printed Films

#### 4.4.1 Tensile Strength Measurements

Three types of composites were prepared to determine the tensile strength of the 3D-printed sheets. The materials consisted of: (a) NFC + alginate; (b) alginate only; and (c) NFC + alginate + cyanobacterial suspension (2.4 g_DCW_ L^–1^). The films were printed to achieve a thickness of 1–2 mm into a glass Petri dish with two layers in a grid pattern (i.e., horizontal and vertical layers) with an edge length of 86 mm. The sheets were cross-linked by submerging in a solution of CaCl_2_ (100 mM) and NaCl (75 mM) for 30 min. The alginate sheet was printed with a reduced *z*-offset to improve adhesion to the glass surface and was cross-linked by immersing in CaCl_2_ as previously mentioned. After cross-linking, the sheets were transferred to a 5 mM CaCl_2_ storage solution and stored for 4 days under constant agitation (60 rpm). Standardized dog-bone specimens were punched out of the 3D-printed specimens. The tensile tests were carried out using a Shimadzu AGS-X universal mechanical testing machine with a speed of 50 mm min^–1^ (Figure S2 and Table S2).

#### 4.4.2 Light Microscopic Imaging

Optical light microscopy was performed in the wet state from freshly mixed ink placed between two glass slides using a Panthera Tech Mat (Motic, China) light microscope in transmittance mode.

#### 4.4.3 Residence Time Distribution

The RTD of the reactors was determined using a step-input method, monitoring the conductivity. A solution of CaCl_2_ (5 mM in water) served as the base fluid, and a tracer solution containing 5 mM CaCl_2_ with 100 mM NaCl was introduced in a step change delivered by a syringe pump at a flow rate of 0.8 mL min^–1^. Conductivity was recorded every 3 s using a conductivity probe (Mettler Toledo InLab 731 ISM) at the reactor outlet using a self-built flow setup (Figure S8) printed out of high-clear resin. The RTD was characterized through step input experiments, and the cumulative distribution function *F*(t) as well as the mean residence time and the dimensionless time were obtained from the conductivity data. The Bodenstein number was calculated using the variance and the variance in dimensionless time (Table S4).

#### 4.4.4 Photosynthetic Activity

The fitness of the cells after 3D printing was determined by measuring the effective yield of Photosystem II (Y­(II)) using an AquaPen-C AP-C 100 hand-held fluorometer (Photon Systems Instruments, Czech Republic). Samples (ca. 1 cm × 3 cm) were inserted into the cuvette, and the light-adapted state of Y­(II) was achieved by applying a strong light pulse (3000 μmol photons m^–2^ s^–1^) on top of the actinic light (50 μmol photons m^–2^ s^–1^) background.

#### 4.4.5 Oxygen Evolution

The light-induced oxygen evolution of the 3D-printed films was determined by measuring the amount of dissolved oxygen in the liquid medium using a robust oxygen probe (OXROB10, Pyroscience, Germany). The measurements were performed 1 day after printing to allow acclimatization of the cells. The films were placed in a glass vial containing BG-11 (4 mL) and subsequently placed in a water bath set at 30 °C. The vials were illuminated by an LED lamp, delivering a light intensity of 250 μmol photons m^–2^ s^–1^. Oxygen evolution was monitored for 15 min, with the first 3 min in the dark. All dissolved oxygen data were processed using the Pyroscience Workbench software.

### 4.5 Whole-Cell Biotransformations and Analytics

After printing, the films were allowed to incubate overnight in BG-11 containing 5 mM CaCl_2_ under a light intensity of 50 μmol photons m^–2^ s^–1^ and stirring (60 rpm). Batch reactions were performed by placing the films in a glass vial containing 5 mM of the substrate solution in 5 mL of BG-11. The reaction is supplemented with 5 mM CaCl_2_ to aid in maintaining the rigidity of the film structure. The vials were then incubated at 30 °C under a constant light regime of 100 μmol of photons m^–2^ s^–1^. Samples (100 μL) were taken periodically, quenched in liquid nitrogen, and stored at −20 °C prior analysis. Three optical densities were tested (OD_750_ = 4, 7, and 10 corresponding to 0.96, 1.68, and 2.4 g_DCW_ L^–1^, respectively) in the whole-cell biotransformation of **1a**. Using the optimized cell density in batch, reactions were then translated to the bioreactor (Figure 5A). The bioreactor setup is shown in Figure S7 consisting of a water bath set at 30 °C, reservoir containing the substrate solution, and a peristaltic pump operated at 0.8 mL min^–1^ to recirculate the solution. An LED growth panel was situated above the reactor to deliver a constant light intensity of ca. 90 μmol photons m^–2^ s^–1^ measured using a LI-COR photometer (LI-250A). Aliquots were taken at the outlet and treated similarly to the batch reactions.

The concentration of substrates and products was determined by a Gas Chromatography (GC) system equipped with a Flame Ionization Detector (FID) (GC-2010 Plus, Shimadzu, Japan), as previously described.^16^ Samples were extracted with ethyl acetate containing *n*-decanol (2 mM) as the internal standard. Quantification of the compounds was performed using a calibration curve obtained for both compounds (Figure S10). Detailed sample preparation steps prior to GC-FID measurements can be found in the Supporting Information (Table S5).

### 4.6 Chlorophyll Determination

The chlorophyll *a* (chl*a*) content of the 3D-printed films was determined using methanol extraction as described previously.^14,26^ Briefly, the films were immersed in 90% (v/v) MeOH in the dark at 60 °C for 60 min. For the bioreactors, three representative films having dimensions of 23 × 8 × 1 mm were 3D-printed and weighed. The absorbance at 665 nm was recorded, and the chl*a* content was determined using a molar extinction coefficient of 78.74 L g^–1^ cm^–1^.

### 4.7 Other Immobilization Techniques

*Synechocystis* sp. PCC 6803 harboring the *yqjM* gene was also immobilized in thin alginate films. For entrapping the cells in thin alginate films, an insect screen (Tesa Insect Shop, Standard 1.30 cm × 1.50 m) was utilized as a support material. Briefly, a mixture of 4% (w/v) alginate and cyanobacterial cells with a starting OD_750_ = 30 was prepared using 1:1 (v/v) ratio with constant stirring for at least 15 min. Afterward, the mixture was pipetted on top of the insect screen and flattened using a glass pipet to distribute the cells uniformly. The film was then sprayed generously with CaCl_2_ (50 mM) to initiate polymerization and left for at least 15 min to harden. Another layer of CaCl_2_ was added to cover the film and left for another 15 min. After cross-linking, the film was cut to the required measurement (1 cm × 3 cm) and washed twice with ddH_2_O prior to usage.

### 4.8 Sustainability Metrics

The AE and related parameters were calculated as detailed by McElroy et al.^66^ The E-factor, on the other hand, was calculated based on equations defined by Sheldon.^67^ The GWP was calculated using equations described by Domínguez de Maria.^73^

## Supplementary Material



## Notes

Abbreviations, general procedure in preparation of the bioink, mechanical tests, raw data for films, RTD, compound retention in the bioreactor, and enantiomeric excess (PDF)

## ∥

School of Engineering Sciences in Chemistry, Biotechnology and Health Science, Science for Life Laboratory, KTH-Royal Institute of Technology, Tomtebodavägen 23a, 171 65 Solna, Schweden Stockholm, Sweden
