# Supplementary material for: Bioprinting of Nanocellulose Hydrogels for Photobiocatalysis Under Continuous Flow
Source: ACS Sustain Chem Eng. 2026 Apr 1;14(14):6599–612. doi: 10.1021/acssuschemeng.5c09689 (PMC13081222; doi:10.1021/acssuschemeng.5c09689)
Supplement: Supplementary file 1 [file sc5c09689_si_001.pdf]

Supplementary Information

Bioprinting of Nanocellulose Hydrogels for Photobiocatalysis Under Continuous Flow

Lenny Malihan-Yap,[a, ‡] Lisa Schmedler,[b,c‡] Daniel Pint,[c,‡] Hitesh Medipally,[a] Florian Lackner,[c] Simon Fedrigotti,[a] Rupert Kargl,[c] Karin Stana Kleinschek,[c,*] Robert Kourist[a,*] and Heidrun Gruber-Woelfler[b,*]

Affiliations

[a]Institute of Molecular Biotechnology, Graz University of Technology, Petersgasse 14, 8010 Graz, Austria

[b]Institute of Process and Particle Engineering, Graz University of Technology, Inffeldgasse 13, 8010 Graz, Austria

[c]Institute of Chemistry and Technology of Biobased System, Graz University of Technology, Stremayrgasse 9, 8010 Graz, Austria

E-mail: karin.stanakleinschek@tugraz.at; kourist@tugraz.at; woelfler@tugraz.at

‡-co-first authors

*co-corresponding authors

Number of Pages: 20

Number of Figures: 13

Number of Tables: 6

Table of Contents

Abbreviations 3

I. Workflow in the preparation of the bio-ink used for batch experiments 4

II. Mechanical tests for the biocomposites 5

III. Oxygen production in the 3D-printed films 6

IV. Effect of shaking on product formation rates 8

V. Stability and fidelity of the 3D-printed films over the course of the reaction 9

VI. Preparation of the bioreactor for continuous flow biotransformation 10

VII. Whole-cell biotransformation of 1a in the 3D-printed bioreactor 11

VIII. Residence time distribution determination 12

IX. Gas chromatography analysis 14

X. Compound retention in the bioreactor 15

XI. Enantiomeric excess determination (%ee) 17

XII. Determination of E-factor 18

XIII. Global warming potential 19

References 20

Abbreviations

**1a**	2-methylmaleimide
**1b**	2-methylsuccinimide
3D	Three-dimensional
ALG	Alginate (sodium alginate)
Bo	Bodenstein number
Chl*a*	Chlorophyll *a* content
DLP	Digital light processing
DCW	Dry cell weight
GGMMA	Galactoglucomannan-methacrylate
NADPH	Nicotinamide adenine dinucleotide phosphate
NFC	Nanofibrillated cellulose
OD	Optical density
PEI	Polyethyleneimine
PETG	Polyethylene terephthalate glycol
RTD	Residence time distribution
SSPCF	Solid-state photosynthetic cell factories
STY	Space-time yield
Synechocystis sp.	*Synechocystis* sp. PCC 6803
TEMPO	2,2,6,6-tetramethylpiperidine-1-oxyl radical
TCNF	TEMPO-oxidized cellulose nanofiber
Y(II)	Effective yield of Photosystem II

I. Workflow in the preparation of the bio-ink used for batch experiments

Cells are harvested and concentrated to an OD_{750}=30, mixed with NFC/ALG and homogenized to produce the bio-ink. Various initial cell densities (*i.e.* 0.24-2.4 g_{DCW} L^{-1}) were combined with the NFC/ALG mixture. The bio-ink was then extruded from a nozzle and 3D-printed into film strips with a thickness of 0.5 mm or 1.0 mm.

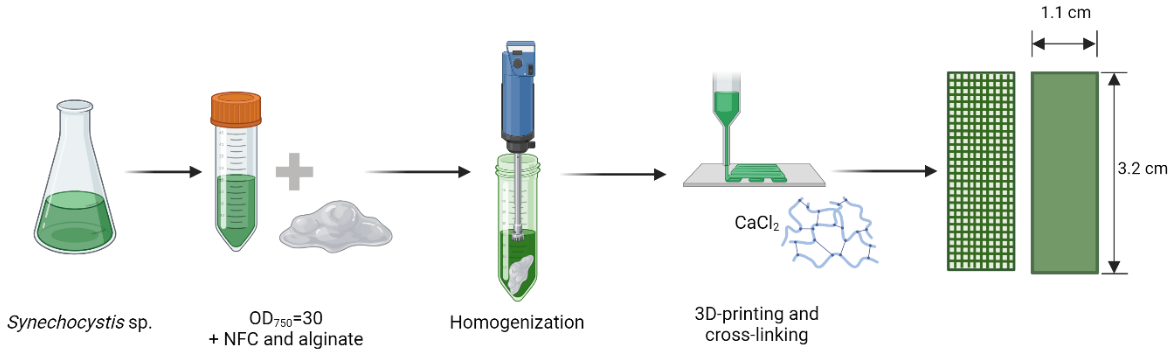

Figure S1. Preparation of bio-ink and 3D-printing. Two thicknesses were prepared for the full-print materials, *i.e.* 0.5 and 1.0 mm.

Table S1. Print settings and dimensions of the films utilized for batch reactions.

Parameter	Full (0.5 mm thick)	Full (1 mm thick)	Mesh
Dimensions [mm^3]	32 x 11 x 0.5	32 x 11x 1	32 x 11 x 1
Infill Distance [mm]	0.53	0.53	1.2
Layers	1	2	2
Thickness [mm]	0.5	1.0	1.0

II. Mechanical tests for the biocomposites

Table S2 shows the compositions of the bioinks tested in this study. To be considered valid, the sample had to fracture within the intended area (*i.e.* at the center) (Figure S2D).

Table S2. Compositions of the biocomposites tested in this study.

Sample	NFC, wt%	Alginate, wt%	OD_{750} Cyanobacteria
NFC/ALG	3	6.65	--
ALG	--	6.65	--
NFC/ALG/Cyano	3	6.65	10

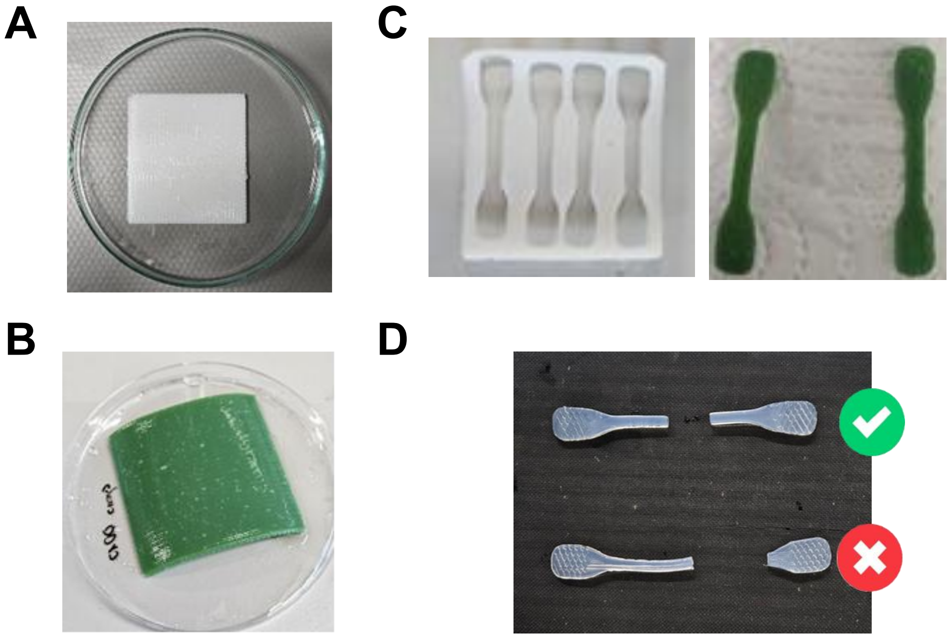

Figure S2. Production of tensile test samples. (A) NFC sheet after 3D-printing; (B) NFC/ALG/CYANO sheet after crosslinking; (C) NFC tensile test cut-outs printed in grid mode and (D) Example of a valid and invalid test

III. Oxygen production in the 3D-printed films

The oxygen release from *Synechocystis* sp. wild-type (WT) entrapped in 3D-printed films were monitored for 15 minutes. The first 3 minutes were in the dark and the oxygen concentration was recorded in light for 12 minutes. Figure S3 shows the representative oxygen production for each cell densities and geometries. Table S3 shows the representative calculation of the rate of oxygen release plotted in Figure 3D.

Table S3. Rate of oxygen production in *Synechocystis* WT entrapped in 3D-printed films.

**Cell density**	**Geometry**	**O_2 production, µmol L^{-1} s^{-1}**	**R^2**	**O_2 production µmol L^{-1} s^{-1} $mg_{chl\textit{a}}^{-1}$ cm^{-2}**
OD 1	1 layer-0.5 mm	-0.0036	0.9507	-0.8674
	2 layers-1.0 mm	-0.0023	0.6580	-10.0468
	2 layers-Mesh	-0.0063	0.8687	-0.8925
OD 5	1 layer-0.5 mm	0.0137	0.9933	6.3015
	2 layers-1.0 mm	0.0306	0.9080	9.2087
	2 layers-Mesh	0.0317	0.9414	7.7070
OD 10	1 layer-0.5 mm	0.0474	0.9964	7.2601
	2 layers-1.0 mm	0.0642	0.9961	7.3421
	2 layers-Mesh	0.0442	0.9809	4.1582

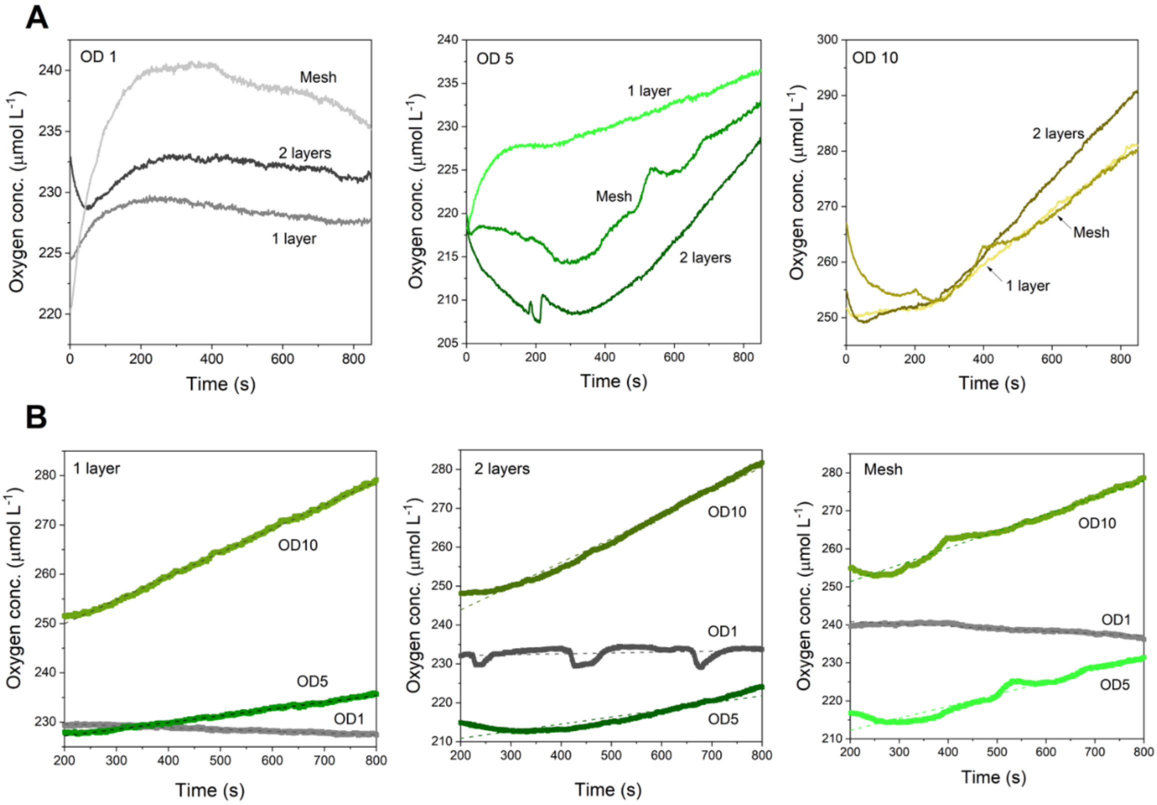

Figure S3. Oxygen production from WT *Synechocystis* sp. immobilized in 3D-printed films at (A) various cell densities and (B) various geometries.

IV. Effect of shaking on product formation rates

The effect of shaking on product formation rates were tested by performing the reactions without agitation. The rates were compared with vials shaken at 140 rpm.

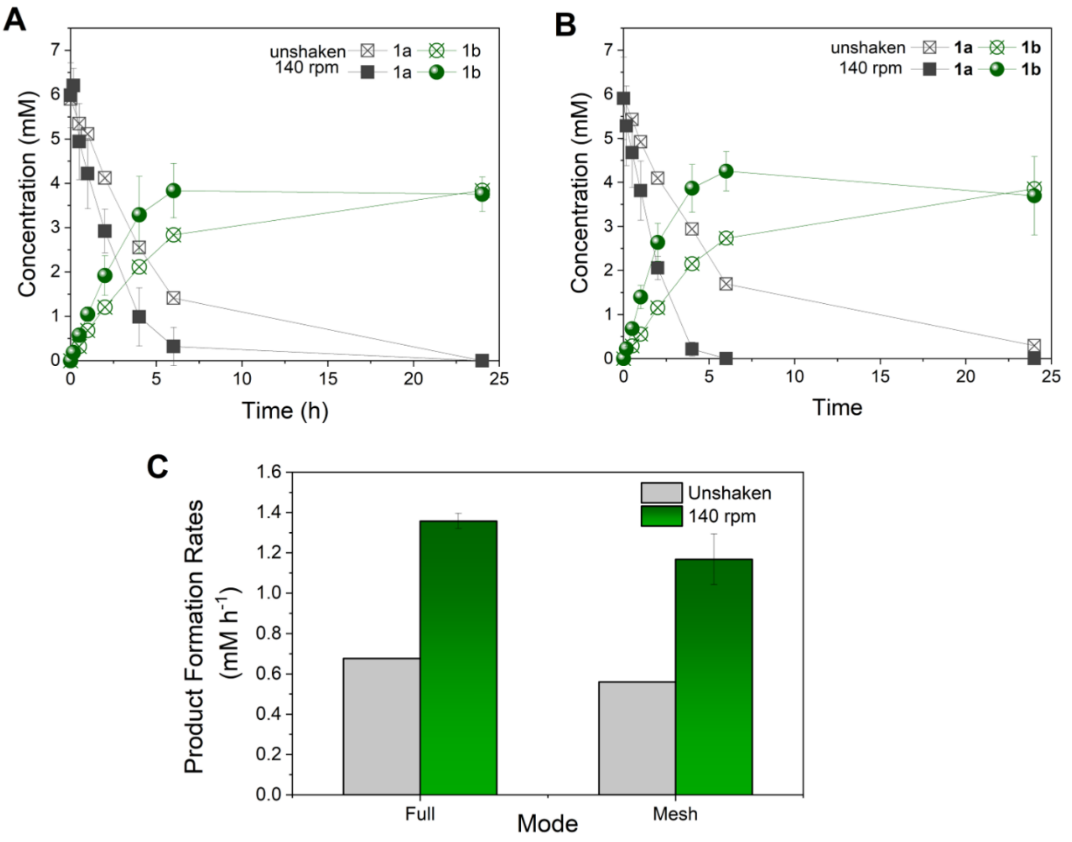

Figure S4. Effect of shaking on the YqjM-mediated biotransformation of **1a** in 3D-printed recombinant *Synechocystis* sp. PCC 6803 in batch. Progress of reaction in (A) Full and (B) Mesh geometries and (C) Comparison of Product formation rates between shaken and unshaken batch reactions.

V. Stability and fidelity of the 3D-printed films over the course of the reaction

The films were formulated as detailed in the Materials and Methods section. Afterwards, they were each placed in a glass vial containing BG-11 (5 mL). The vials were then placed on a rotary shaker set at 140 rpm. Photos were taken after 2, 4 and 24 hours to illustrate the stability and fidelity of the films.

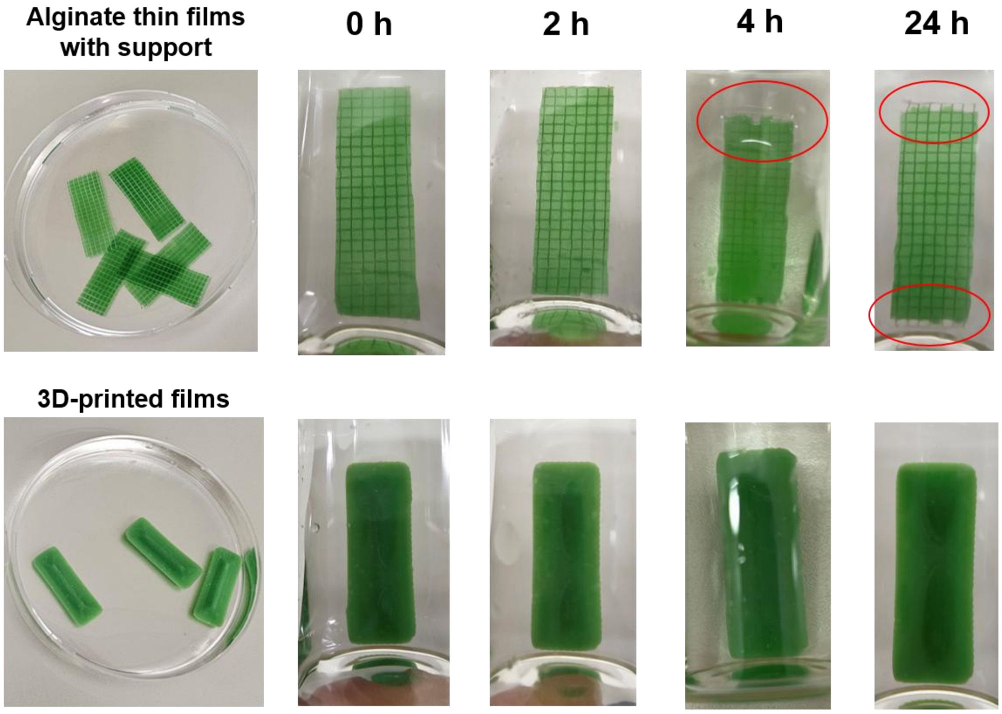

Figure S5. Investigating the stability and fidelity of immobilized *Synechocystis* sp. entrapped in alginate-based formulations. *Reaction conditions*: BG-11, 140 rpm

VI. Preparation of the bioreactor for continuous flow biotransformation

The Plexiglas was first treated in a UV-ozone system for 15 min to oxidize the surface followed by treatment with PEI (5%). The bottom plate was initially designed with a negative anchor profile to hold the ink. However, this showed too much swelling and the anchor profile was finally attached using surface coating. Figure S6 shows the cross-sectional view of the 3D-printed bottom plate with the anchor profile and the 3D-printed scaffold.

(A)

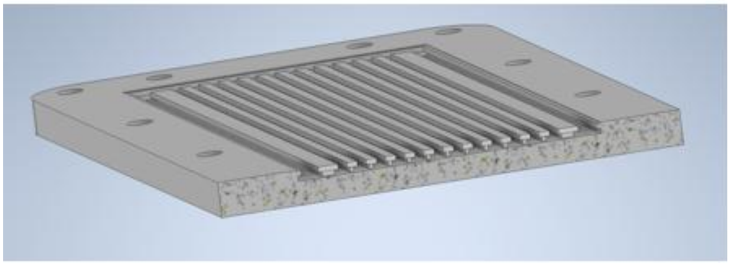

(B)

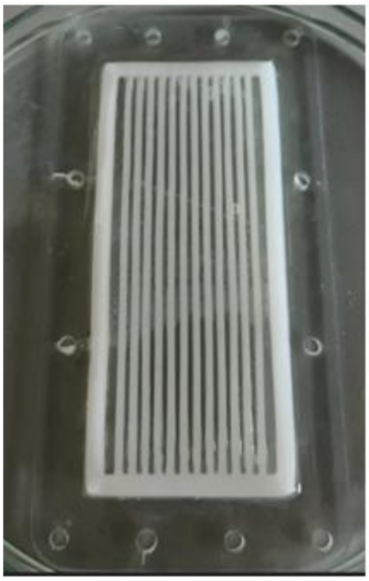

(C)

	Static crosslinking	Shaking crosslinking[a]	Shaking storage[b]
**Anchor profile**	✓	✓	✓
**PEI coating**	✓	✓	✓

[a]90 rpm for 30 min; [b]0.9 wt% NaCl, 47 rpm overnight

Figure S6. Design for the 3D-printed reactor. (A) Cross-sectional view of the 3D-printed bottom plate with the negative anchor profile from the .stl file; (B) Crosslinked scaffold and (C) Testing conditions for fixation method and storage.

VII. Whole-cell biotransformation of 1a in the 3D-printed bioreactor

The 3D-printed bioreactors were placed below a light source and the substrate was delivered using a peristaltic pump. The solution containing the substrate was recycled to the reactor for 24 h. Samples were taken at the outlet and analysed by GC-FID.

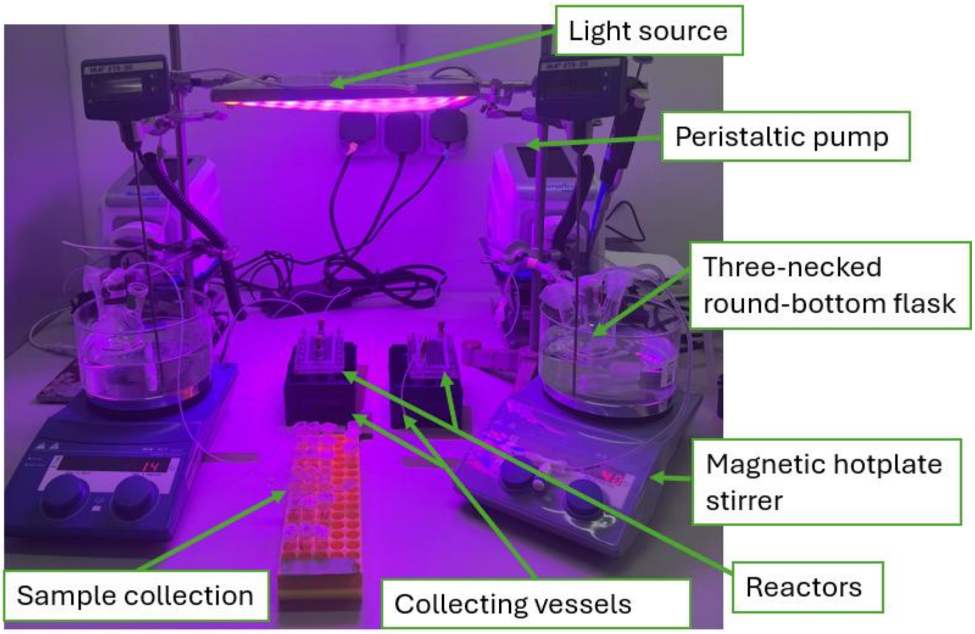

Figure S7. Reaction set-up for the biotransformation of **1a** using the 3D-printed bioreactors.

VIII. Residence time distribution determination

The residence time distribution (RTD) and flow behavior of the string and line reactors was analysed using conductivity measurements. Traditional methods using a dye were not feasible due to partial disintegration of the bioink.

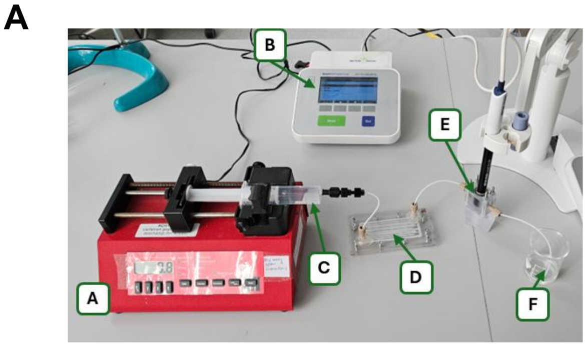

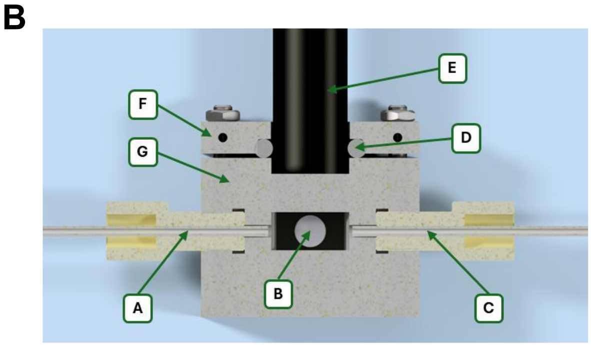

Figure S8. (A) Experimental set-up for the RTD tests and the (B) self-built flow cell set-up for residence time distribution by monitoring the conductivity of the tracer solution containing 5 mM $CaCl_2$ and 100 mM NaCl.

The following equations were utilized to determine the residence time and other flow parameters.

Table S4. Residence time distribution results for the String and Line Reactor.

Parameter	Symbol	Equation	String	Line
Cumulative distribution function	$F_{(t)}$	$F_{(t)} = \frac{c_i}{c_{max}}$		
Mean residence time	$\bar{t}$	$\bar{t} = \frac{\sum t_i \times \Delta c_i}{c_{max}}$	233 s	813 s
Dimensionless time	θ	$\theta = \frac{t_i}{\bar{t}}$		
Variance	σ^2	$\sigma^2 = \frac{\sum t_i^2 \times \Delta c_i}{c_{max}} - \bar{t}^2$	33189	484357
Bodenstein Number	*Bo*	$Bo = \frac{2}{\sigma_\theta^2}$	3.26	2.73
Bodenstein Number for *Bo* < 100	Bo_{oo}	$Bo_{oo} = \frac{1}{\sigma_\theta^2} + \sqrt{\left(\frac{1}{\sigma_\theta^2} + \frac{8}{\sigma_\theta^2}\right)^2}$	5.59	4.94

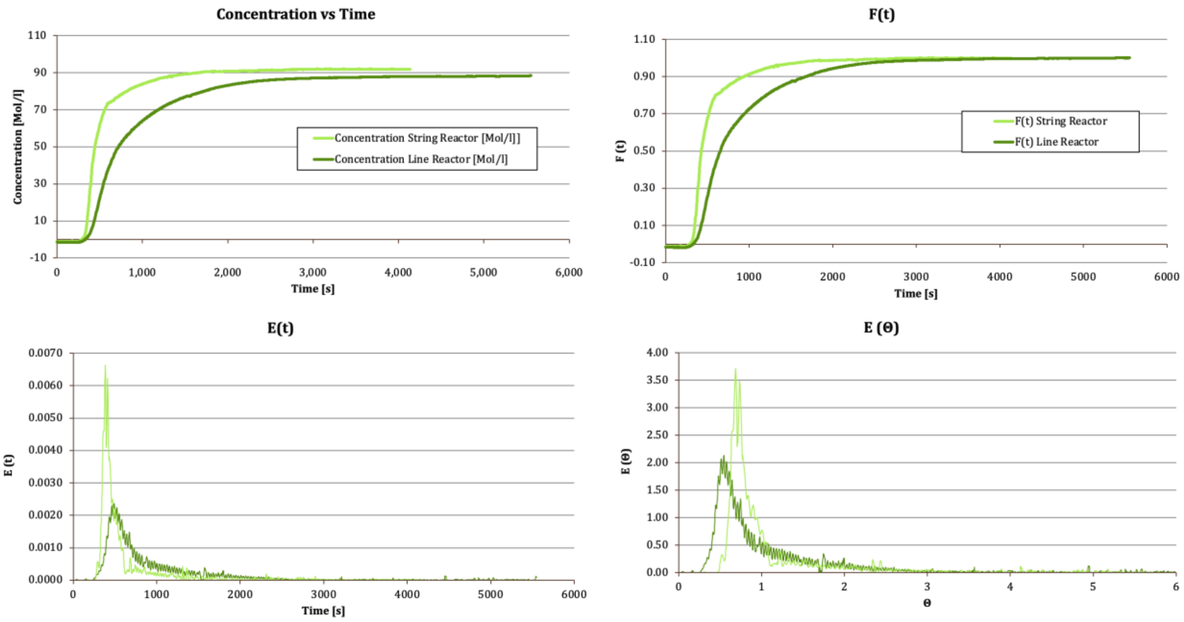

Figure S9. Comparison of residence time distribution results for the Line and String reactors.

IX. Gas chromatography analysis

Quantitative analysis of the compounds **1a** and **1b** was performed using gas chromatography with a flame ionization detector (GC-FID, GC2010 Plus, Shimadzu, Japan) fitted with an achiral ZB-5 column (film thickness= 0.25 μm, column length= 30 m, inner diameter= 0.32 mm) having a stationary phase of 5% phenyl and 95% dimethylpolysiloxane. Sample was prepared using organic phase extraction with ethyl acetate containing 2 mM *n*-decanol as internal standard. Briefly, samples (100 μL) were extracted with the extraction solvent (300 μL) by inverting up and down for 1 minute. The organic phase was dried using a spatula tip of anhydrous $MgSO_4$ and finally vortexed prior to GC analysis. Table S5 shows the GC-FID parameters utilized and Figure S10 show the calibration standards for both 1a and 1b.

Table S5. GC-FID method for the determination of **1a** and **1b**.

Parameters		
**Autosampler and Injection Port**	Injection volume	1 μL
	Injection Temp.	230 ºC
	Carrier Gas	N_2
	Total Flow	19.8 mL min^{-1}
	Column Flow	0.80 mL min^{-1}
	Linear Velocity	17.6 cm s^{-1}
	Purge Flow	3 mL min^{-1}
	Split Ratio	20
**Temp. program**	100 ºC, hold for 3 min;<br>310 ºC, hold for 4 min with a rate of 30 ºC min^{-1}	
**FID detector**	Temperature	320 ºC
	Sampling rate	40 ms
	H_2 flow	40 mL min^{-1}
	Air flow	400 mL min^{-1}

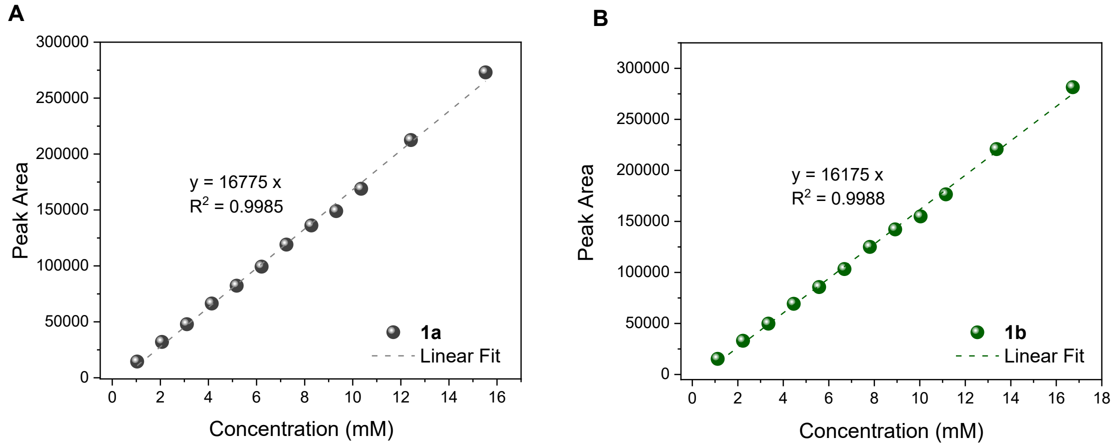

Figure S10. Calibration standards used to determine (A) **1a** and (B) **1b** concentration during biotransformation.

X. Compound retention in the bioreactor

The immobilized catalyst was scraped off from the Plexiglas and support and extracted with ethyl acetate. The suspension was allowed to incubate in ethyl acetate for 4 hours. Afterwards, the supernatant was analysed using GC-FID. Figure S11 shows the chromatogram after extraction. The product **1b** was retained on the immobilized catalyst.

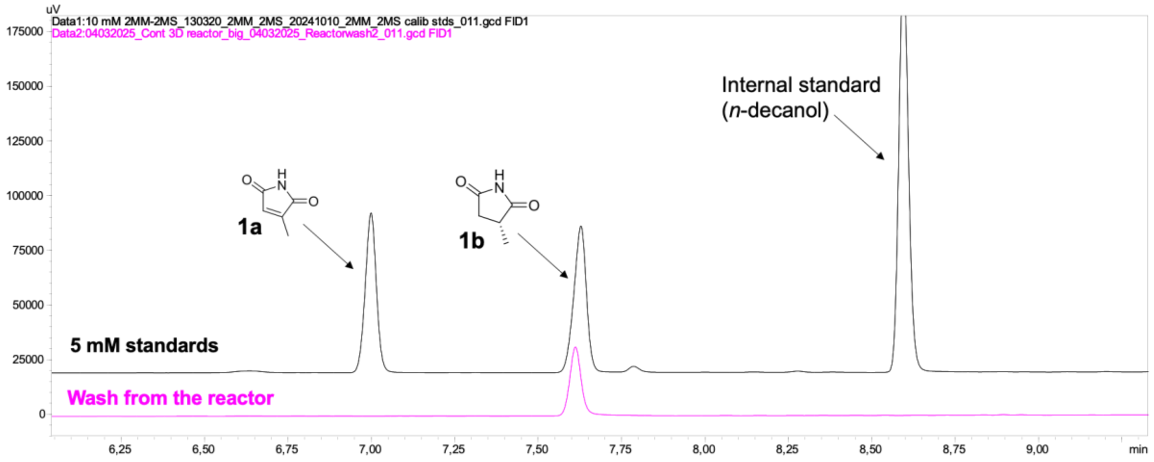

Figure S11. GC-FID analysis of the extract obtained from the line reactor after the 24h reaction.

A ‘blank’ line reactor (9 x 3 cm) was built without addition of the cells to assess the retention capability of the polymer. The reaction was performed with either pure **1a** or pure **1b** and the change in concentration was determined by GC-FID. Figure S12 shows the time profile for the compounds during the reaction.

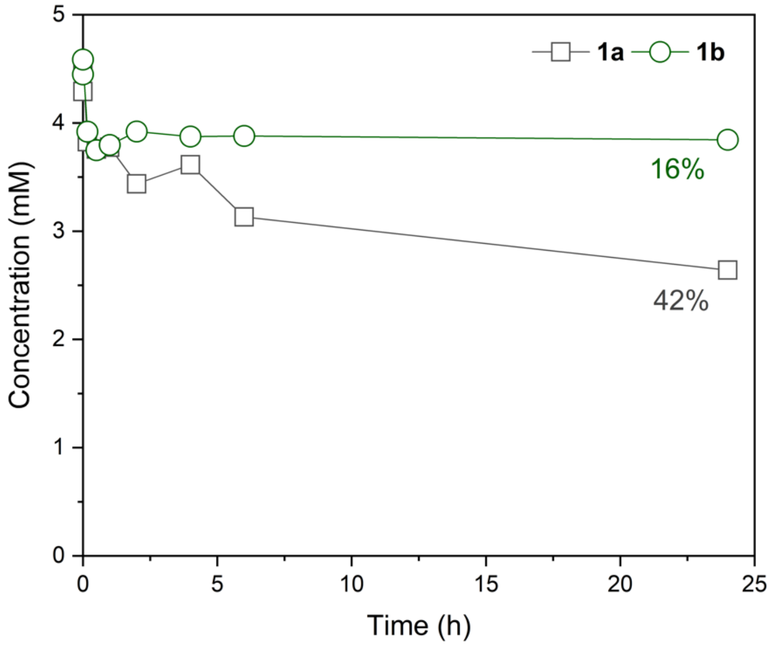

Figure S12. Time profile of concentration change in the line reactor without *Synechocystis* cells using pure compounds. *Reaction conditions:* 0.8 mL min^{-1}, 5 mM **1a** or **1b**, BG-11 (+5 mM $CaCl_2$), 100 µmol photons m^{-2} s^{-1}, line reactor (3 x 9 cm), *N*= 1

XI. Enantiomeric excess determination (%ee)

The enantiomeric excess of the reaction was determined by injecting the 24 h sample in GC-FID outfitted with a chiral column (β-6TBDAc). The column has a length of 50 m, film thickness of 0.25 µm and a diameter of 0.25 mm as previously described. [1,2] As a reference, a racemate standard of the product **1b** (5 mM in BG-11) was also injected.

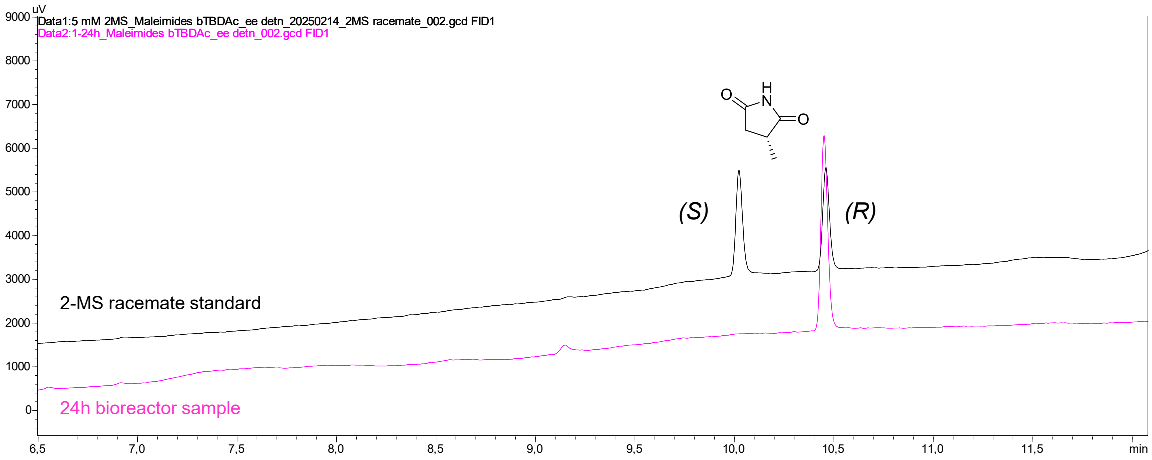

Figure S13. GC-FID chromatogram of a 24h sample from the line reactor compared with a standard of *rac*-2-methylsuccinimide.

XII. Determination of E-factor

The E-factor defined as the amount of total wastes generated over the amount of product was calculated based on the parameters detailed in Table S6. The E-factor was calculated at an operating time of 4 h for all studies. Hence, parameters for other published works [1–3] were re-calculated based on available data.

Table S6. Parameters for the calculation of the E-factor for **1a** reduction using recombinant *Synechocystis* sp. harbouring the *yqjm* gene from *B. subtilis* in various reactor concepts.

Parameter	Immobilized reactors				Other works		
	Full	String	Line	Continuous	Flat panel[3]	Coil[1]	BCR[2]
Reaction volume, mL		20		10.4	120	15	200
Operating time, h				4			
Medium ingredients, g		0.06		0.03	0.34	0.06	0.82
Water, g		19.94		10.31	119.57	14.95	198.70
Cells, g_{DCW}	0.006	0.003	0.005	0.019	0.432	0.054	0.480
**1a** fed, g		0.011		0.012	0.667	0.067	0.889
**1b** formed, g	0.005	0.006	0.009	0.008	0.413	0.052	0.329
sEF[a]	12.80	11.73	7.23	6.99	2.48	2.50	5.65
cEF[b]	3731	3584	2263	1383	292	290	610

[a]sEF refers to the simple E-factor excluding water in the calculation; [b]cEF refers to complete E-factor including wastewater

XIII. Global warming potential

The global warming potential (GWP) of the reaction was calculated based on Equations [1] and [2] expressed as kg CO_2 per kg product as described.[4]

$$\mathrm{GWP(water(energy))} = \left(\frac{0.00037 \cdot \Delta T}{[P]}\right) + t \cdot \left(\frac{0.000056 \cdot \Delta T}{[P]}\right) \qquad [1]$$

For the proposed downstream processing using ethyl acetate extraction, the following equation was utilized,

$$\mathrm{GWP(extraction(dsp))} = \left(\frac{(0.0002 \cdot n \cdot \Delta T) + (0.00008 \cdot n \cdot \Delta H)}{[P]}\right) + \left(\frac{(0.021 \cdot n \cdot \%\mathrm{SolventTreated})}{[P]}\right) + \left(\frac{(f \cdot \%\mathrm{WaterTreated})}{100 \cdot [P]}\right) \qquad [2]$$

Where: ΔT is the change of temperature from 20 °C to the reaction temperature in °C; [P] is the product titer in kg L^{-1}; n is the number of extraction times; ΔH is the enthalpy of vaporization of the extraction solvent in kJ kg^{-1} and f is a factor depending on the type of effluent (0.073 for mild wastewater treatment plants)

References

(1) Valotta, A.; Malihan-Yap, L.; Hinteregger, K.; Kourist, R.; Gruber-Woelfler, H. Design and Investigation of a Photocatalytic Setup for Efficient Biotransformations Within Recombinant Cyanobacteria in Continuous Flow. *ChemSusChem* **2022**, *15* (22), 1–11. https://doi.org/10.1002/cssc.202201468.

(2) Hobisch, M.; Spasic, J.; Malihan-Yap, L.; Barone, G. D.; Castiglione, K.; Tamagnini, P.; Kara, S.; Kourist, R. Internal Illumination to Overcome the Cell Density Limitation in the Scale-up of Whole-Cell Photobiocatalysis. *ChemSusChem* **2021**, *14* (15), 3219–3225. https://doi.org/10.1002/cssc.202100832.

(3) Grimm, H. C.; Erlsbacher, P.; Medipally, H.; Malihan-Yap, L.; Sovic, L.; Zöhrer, J.; Kosourov, S. N.; Allahverdiyeva, Y.; Paul, C. E.; Kourist, R. Towards High Atom Economy in Whole-Cell Redox Biocatalysis: Up-Scaling Light-Driven Cyanobacterial Ene-Reductions in a Flat Panel Photobioreactor. *Green Chem.* **2025**, *27*, 2907–2920. https://doi.org/10.1039/d4gc05686h.

(4) Domínguez de María, P. (Re)Visiting the Sustainability Thresholds: Are Product Titers of 1 g L^{-1} Enough for (Bio)Chemical Processes? *ChemSusChem* 2025. https://doi.org/10.1002/cssc.202501831.
